# High abundance of pyrrolizidine alkaloids in bee pollen collected in July 2019 from Southern Germany

**DOI:** 10.1007/s10661-022-09907-8

**Published:** 2022-03-06

**Authors:** Carolin Friedle, Thomas Kapp, Klaus Wallner, Raghdan Alkattea, Walter Vetter

**Affiliations:** 1 Apicultural State Institute, University of Hohenheim, Stuttgart, Germany; 2 Chemical and Veterinary Analysis Agency (CVUA), Stuttgart, Fellbach, Germany; 3 Institute of Food Chemistry (170B), University of Hohenheim, Stuttgart, Germany

**Keywords:** Pyrrolizidine alkaloids, Bee-collected pollen, Palynological analysis, LC–MS/MS, Risk management

## Abstract

Pyrrolizidine alkaloids (PA) are secondary plant defense compounds and known pre-toxins when containing a 1,2-double bond. They are commonly produced by various plants and may thus be present in bee pollen which may be consumed by humans as food supplements. In this study, PA were determined in bee pollen samples from 57 locations in Southern Germany sampled by means of pollen traps in July 2019. Samples were analyzed by using palynological methodology and solid-phase extraction (SPE) followed by LC–MS/MS. In total, 52 pollen samples featured total pyrrolizidine alkaloids (ΣPA) with concentrations up to 48,000 ng/g bee pollen, while the N-oxides (NO) echinatine-NO and rinderine-NO clearly dominated. In contrast, the palynological analysis only detected 33 samples with pollen from PA-producing plants. Accordingly, the results showed that palynological analysis is not sufficient to determine PA in pollen. In addition, a risk assessment was followed to estimate the risk of the detected PA concentrations to humans.

**Supplementary information:**

The online version contains supplementary material available at 10.1007/s10661-022-09907-8.

## Introduction

From early spring to late summer, honey bees (*Apis mellifera*) are collecting pollen from various plants. Being rich in protein, fatty acids, and vitamins, bee pollen is the primary nutritional source for bees (Avni et al., 2014; Mărgăoan et al., 2014; Taha et al., 2019). Due to these valuable ingredients, bee pollen is also an attractive food supplement in human nutrition (Feás et al., 2012). For this purpose, bee pollen can be collected by the installation of pollen traps at the hive from early spring (usually April to June) to late summer. However, a thorough control of samples is important because bee pollen may be contaminated with both residues of pesticides applied in agriculture mainly during spring (April to June) (Böhme et al., 2018; Drummond et al., 2018; Friedle et al., 2021; Traynor et al., 2016) and also harmful natural contaminants produced by plants during summer (June to August) (BfR, 2013).

**Fig. 1:**
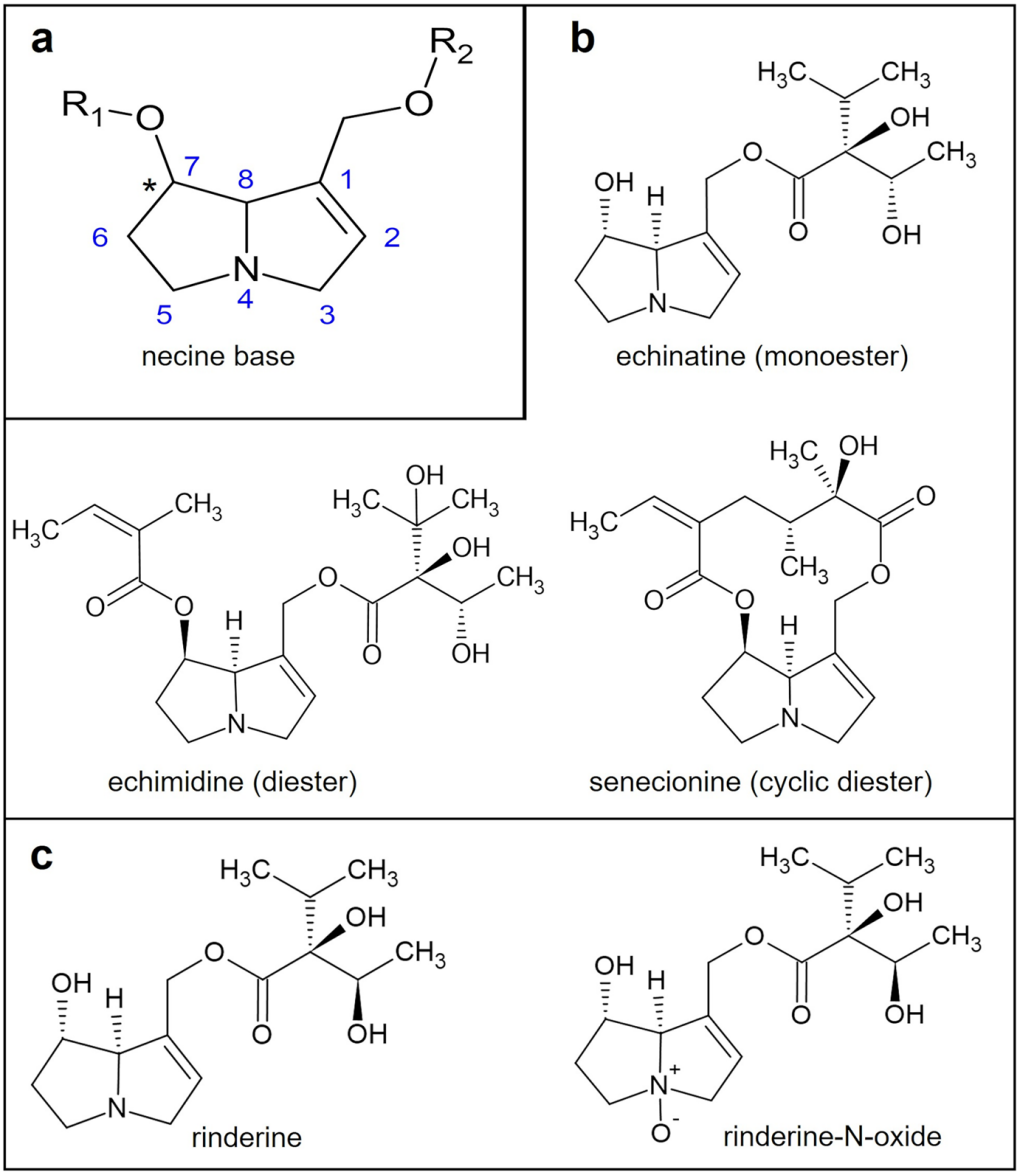
**a** Structures of necine base. **b** Examples of PA with different kinds of esterification. **c** A PA structure (rinderine) with the corresponding N-oxide (PANO)

One class of potential natural contaminants of bee pollen is pyrrolizidine alkaloids (PA) (EFSA, 2011, 2017). PA are a group of secondary plant defense compounds whose common structural element is a 1-azabicyclo[3.3.0]octane (pyrrolizidine) backbone which usually carries an additional 1,2-double bond (present in all toxic variants) along with a hydroxymethyl substituent in the 1-position and a hydroxyl group in the 7-position, respectively (Fig. 1a). The resulting PA core is the so-called necine base which is either esterified once (monoesters) or twice (diesters or cyclic diesters) with acyl moieties varying in structure and stereochemistry (Fig. 1b). Likewise, the stereocenter in 7-position (*) can be both *R-* or *S*-configurated (Fig. 1a). Last but not least, virtually all PA exist in two distinct forms, e.g., tertiary heterocyclic amines and the corresponding N-oxide form (PANO) (Fig. 1c). These structural variations give rise to more than 600 structurally different PA (EFSA, 2011). In the following, the designation “PA” is also used as summarizing term for PA and PANO. Assumedly, around 3% of all flowering plants may produce PA (Smith & Culvenor, 1981). Most of these plants belong to the families of *Asteraceae* (e.g., *Senecioneae* (*Senecio* spp.) and *Eupatorieae* (*Eupatorium* spp.)), *Boraginaceae* (e.g., *Borago* spp. and *Echium* spp.), and *Fabaceae* (e.g., *Crotalaria* spp.) (EFSA, 2011; Hartmann & Toppel, 1987).

Harmful PA are hepatotoxic to both animals and humans and can cause acute poisoning and chronic effects (Colegate et al., 2012; EFSA, 2011; Kakar et al., 2010). Chronic exposure to low PA amounts can result in diseases such as liver cirrhosis or possibly cause cancer as metabolic activation produces genotoxic and cancerogenic metabolites (Edgar et al., 2011). As a consequence, the US Food and Drug Administration (FDA) decreed to ban PA-containing products from the market (Food & Drug Administration, 2001). By contrast, maximum levels of PA are currently not in force for food in the European Union (EU). A novel EU regulation defining maximum PA levels for pollen and pollen-based food supplements among other foods such as (herbal) teas, herbs, and spices will be effective by July 2022. Then, for pollen and related food supplements a maximum PA level of 500 µg/kg must not be exceeded (European Commission, 2020). Furthermore, a maximum intake of 1 µg PA per day was established in pharmaceutical products (Federal Institute for Pharmaceuticals and Medical Products, 2016). Likewise, the European Food Safety Authority (EFSA) has introduced a benchmark dose lower confidence limit 10% (BMDL_10_) amount of 237 µg riddelliine per kg body weight (BW) in rats as a reference point for the assessment of carcinogenic risks, assuming similar carcinogenic potency for different PA (EFSA, 2017). Considering a tolerable margin of exposure (MOE) of 10,000 for humans, this corresponds with a maximum intake of 0.024 µg PA per kg BW per day (BfR, 2020). This in turn corresponds with a maximum daily intake of 1.8 µg PA per day for adults (75 kg BW) and ~1 µg for juveniles (40 kg BW, 10 years) (GBE, 2017).

Next to foods with a direct botanical background such as teas and herbals, PA were also exemplarily studied in honey and bee pollen (Bodi et al., 2014; Boppré et al., 2005; Dübecke et al., 2011; Gottschalk et al., 2018; Kaltner et al., 2020; Kast et al., 2018, 2019; Kempf et al., 2010, 2011; Martinello et al., 2014; Orantes-Bermejo et al., 2013; Roeder, 1995). In one study, 17 of 55 commercial pollen products, mainly from Spain, Romania, Italy, and France, were contaminated with toxic PA of up to 16,400 ng/g pollen (Kempf et al., 2010). Similarly, Dübecke et al. (2011) detected PA in 60% of 119 bee pollen samples from various countries with ΣPA contents of up to 37,900 ng/g pollen. Recently, Kast et al. (2018) presented two complementary methods: a high-performance liquid chromatography coupled to tandem mass spectrometry (LC–MS/MS) method which allowed studying 18 PA and PANO in commercial bee pollen products as well as daily collected bee pollen samples, and a LC-HRMS method comparing daily collected pollen samples to flower heads, by detecting of all PA types, including saturated, non-cancerogenic PA. To achieve higher confidence for identification of the plant source, nearly all PA-containing plants occurring in Switzerland were also analyzed by LC-HRMS. Bee-collected pollen indicated the presence of highest PA concentrations in “*Echium*-type PA” samples collected in June and “*Eupatorium*-type PA” samples (assigned as intermedine and lycopsamine (-N-oxides)) mainly from mid-July and August (Kast et al., 2018). However, no reliable information existed about PA levels in bee-collected pollen from Germany.

Given the geographic neighborhood of Switzerland (the observation site Basel of Kast et al. (2018) is directly at the border to Germany) and Southern Germany, we aimed to carry out a thorough study by collecting bee pollen by means of traps in 57 locations in Baden-Wuerttemberg (Southern Germany). Knowing the fact that pollen samples in spring (from April to June) could be highly contaminated with pesticides in Southern Germany (Friedle et al., 2021), the aim was to determine whether the pollen samples were otherwise contaminated in a later period. In the end of July 2019 (July 15–August 1), bee pollen was daily collected on each site on 14 consecutive days, and then pooled. The pooled site samples were analyzed palynologically using a microscope to get insights into the botanical background of the samples. Chemical analysis was carried out with an optimized LC–MS/MS method which allowed determining 42 PA simultaneously. The results were used to evaluate whether bee pollen from Baden-Wuerttemberg (Southern Germany) can be safely consumed.

## Materials and methods

### Chemical reagents

PA standards used for analysis were 7-acetylintermedine, 7-acetylintermedine-N-oxide, 7-acetyllycopsamine, 7-acetyllycopsamine-N-oxide, echinatine, echinatine-N-oxide, heliosupine, heliosupine-N-oxide, heliotrine, heliotrine-N-oxide, indicine hydrochloride, indicine-N-oxide, integerrimine, integerrimine-N-oxide, intermedine, lycopsamine, lycopsamine-N-oxide, retrorsine, riddelliine, riddelliine-N-oxide, rinderine, rinderine-N-oxide, senecionine, senecionine-N-oxide, seneciphylline, and senkirkine, from PhytoPlan (Heidelberg, Germany). Echimidine, echimidine-N-oxide, erucifoline, erucifoline-N-oxide, europine hydrochloride, europine-N-oxide, intermedine-N-oxide, jacobine, jacobine-N-oxide, lasiocarpine, monocrotaline-N-oxide, retrorsine-N-oxide, seneciphylline-N-oxide, senecivernine, and senecivernine-N-oxide were from PhytoLab (Vestenbergsgreuth, Germany). Lasiocarpine-N-oxide was from Cfm Oskar Tropitzsch (Marktredwitz, Germany). Monocrotaline was from Carl Roth (Karlsruhe, Germany) and trichodesmine from Latoxan (Valence, France). For the palynological analysis and sample preparation, the following reagents were used: demineralized water, ultrapure water, dish soap, Kaiser’s glycerol gelatin, dry ice, 0.05 M sulfuric acid, ammonia solution, and methanol. For sample analysis, 5 mM ammonium formate and 0.1% (v/v) formic acid in methanol were used.

### Sample collection

Bee pollen traps were installed on apiaries at 57 locations in Baden-Wuerttemberg, Southern Germany, to collect pollen loads from returning honey bees (*Apis mellifera*) (Fig. S1). All traps were installed on privately owned bee colonies of voluntary beekeepers, so no exact coordinates will be given and no permits were needed for this study. The collecting time (July 15–August 1) was adapted to the flowering phase of *Borago* sp., *Echium* sp., *Eupatorium* sp., and *Senecio* sp. at the end of July 2019. Pollen samples were collected daily within 14 consecutive days (rainy days uncounted) and mixed to one pooled sample per site. An aliquot of 45 g pooled pollen per site was taken and stored at −20 °C until preparation.

### Sample preparation

Bee pollen samples were prepared following a validated solid-phase extraction (SPE) protocol originally developed by the BfR (2014). In brief, samples were brought up to room temperature and homogenized with dry ice in a mill (Retsch, Haan, Germany). An aliquot of 2.0 g ± 0.1 g bee pollen was weighted into a tube (Sarstedt, Nümbrecht, Germany), mixed with 20 mL 0.05 M sulfuric acid and extracted for 15 min in an ultrasonic bath. The samples were centrifuged (5 min, 3000 × *g*) and the supernatant was transferred to another tube. The pellet was used for a second extraction using 20 mL 0.05 M sulfuric acid and was re-extracted for 15 min in an ultrasonic bath. After centrifugation (5 min, 3000 × *g*) the supernatant was transferred to the first extract and adjusted to pH 6–7 with an ammonia solution. SPE was carried out with DSC-C18 SPE cartridges (500 mg, 6 mL, Supelco Merck, Darmstadt, Germany) in a vacuum chamber as follows. Conditioning was performed using 5 mL methanol, followed by 5 mL ultrapure water. The sample extract was loaded onto the cartridge (2 × 5 mL), followed by washing with 2 × 5 mL ultrapure water and 5 min drying under vacuum conditions. The sample was eluted with 5 mL methanol and evaporated to 1 mL in a heating block maintained at 55 °C under nitrogen flow. After addition of ultrapure water to a total volume of 10 mL, an aliquot was filled into an LC vial for analysis.

### LC–MS/MS analysis of pyrrolizidine alkaloids

PA single component analysis on 42 PA/PANO was performed with a 6490 tandem mass spectrometer (Agilent Technologies, Waldbronn, Germany) coupled to a 1290 ultra-high-performance liquid chromatography (UHPLC) system (Agilent Technologies, Waldbronn, Germany) at the Chemical and Veterinary Analysis Agency (CVUA Stuttgart, Germany). Chromatographic separation was carried out on a 150 mm × 2.1 mm i.d., 1.7 µm particle-sized Acquity CSH C18 UPLC column (Waters, Eschborn, Germany), using aqueous 5 mM ammonium formate solution with 0.1% (v/v) formic acid as eluent A and 5 mM formate solution with 0.1% (v/v) formic acid in methanol as eluent B. Gradient elution started at 2.5% eluent B (1.0 min), linearly increased to 10% eluent B at 15.5 min, and then increased further to 23% eluent B at 20.5 min and in the next step to 36% eluent B at 25.0 min before being ramped to 100% B at 28.0 min (1.5 min). At 29.6 min, initial conditions were restored and kept until the final run time of 31.5 min. Flow rate was set at 0.35 mL min^−1^ at a controlled column temperature of 50 °C. The injection volume was 2 µL for all runs.

The Jet Stream electrospray ion source was operated in positive mode at a capillary voltage of 3500 V. The nebulizer pressure was set to 25 psi with a gas flow of 13 L min^−1^ at a temperature of 250 °C. Sheath gas flow rate was set to 12 L min^−1^ at a temperature of 360 °C. High- and low-pressure ion funnel RFs were set to 150 V and 60 V, respectively. For each analyte, three transitions were recorded in dynamic multiple reaction monitoring (dMRM) mode (Table 1). In dMRM mode, an acquisition window of 6 min was set around the retention time of each PA and the cycle time was fixed at 500 ms. To increase signal intensity, a ∆EMV setting of + 200 V was employed. To avoid matrix effects, an external matrix-matched calibration employing 7-point calibration curves was performed and resulted in limits of detection (LOD) and limits of quantification (LOQ) as shown in Table 1. LOD and LOQ were determined according to DIN 32,645 by calculating the process standard deviation s_x0_ of the linear calibration curve, whereas the LOD was defined as the 3.6-fold value of s_x0_ and the LOQ as the 10.8-fold value of s_x0_, respectively (DIN 32654, 2008). Values below the LOQ are given as not detectable. The validated linear working range of the method was between 0.1 and 40 ng/mL. Sample solutions exceeding this calibration range were diluted accordingly. For method validation, recovery values were determined at low and high spiking levels by spiking a blank fennel matrix with known amounts of each analyzed PA(NO) at levels of 8 and 80 µg/kg plant material in quintuplicate, respectively. For verification purposes of this pollen study, recovery values were determined by spiking a blank pollen matrix with known amounts of each analyzed PA(NO) at a medium to low PA level of 20 µg/kg pollen (Table 1). Throughout the study, sample results were not corrected for recovery.

**Table 1:** Analyzed substances with MRM details. LOD (ng/g), LOQ (ng/g) and recovery rate (%) as obtained by the laboratory (CVUA)

Pyrrolizidine alkaloid	Retention time (min)	Precursor ion (m/z)	Product ion (m/z)	Quantifier	Collision energy (eV)	Cell accel. (V)		LOD (µg/kg)	LOQ (µg/kg)	Recovery rate (fennel matrix 8 µg/kg)	Recovery rate (fennel matrix 80 µg/kg)	Recovery rate (pollen matrix 20 µg/kg)
7-Acetylintermedine	17.87	342.2	198.1		36	1		0.53	1.6	105%	106%	91%
		342.2	180.1		16	3						
		342.2	120.1	x	30	3						
7-Acetylintermedine-N-oxide	19.28	358.2	214.1	x	30	4		0.40	1.2	99%	102%	92%
		358.2	180.1		32	5						
		358.2	137.1		34	1						
7-Acetyllycopsamine	18.30	342.2	198.1		32	4		0.24	0.71	106%	107%	71%
		342.2	180.1		16	5						
		342.2	120.1	x	30	1						
7-Acetyllycopsamine-N-oxide	19.63	358.2	214.1	x	32	1		0.75	2.3	101%	104%	88%
		358.2	180.1		32	1						
		358.2	137.1		38	1						
Echimidine	23.66	398.2	220.1		16	2		0.35	1.1	91%	98%	91%
		398.2	120.1	x	28	1						
		398.2	83.0		28	2						
Echimidine-N-oxide	23.97	414.2	254.1	x	36	2		0.32	0.97	90%	98%	94%
		414.2	220.1		34	3						
		414.2	137.1		40	1						
Echinatine (with rinderine)	7.84	300.2	156.1		28	5		0.09	0.26	99%	103%	90%
		300.2	138.1	x	24	1						
		300.2	94.1		50	3						
Echinatine-N-oxide	10.28	316.2	172.1	x	30	5		0.28	0.84	101%	100%	74%
		316.2	138.1		32	3						
		316.2	94.1		50	3						
Erucifoline	5.65	350.2	322.2		26	1		0.30	0.89	104%	113%	93%
		350.2	120.1	x	30	1						
		350.2	67.1		54	1						
Erucifoline-N-oxide	9.28	366.2	136.1	x	36	1		0.93	2.8	120%	117%	103%
		366.2	120.1		35	4						
		366.2	118.1		38	4						
Europine	8.00	330.2	156.1		34	4		0.14	0.42	104%	104%	91%
		330.2	138.1	x	22	4						
		330.2	120.1		42	4						
Europine-N-oxide	10.05	346.2	256.1		28	1		0.18	0.54	97%	97%	80%
		346.2	172.1	x	36	3						
		346.2	111.1		54	1						
Heliosupine	23.80	398.2	336.2		18	1		0.24	0.72	93%	100%	93%
		398.2	220.1		18	1						
		398.2	120.1	x	30	1						
Heliosupine-N-oxide	25.64	414.2	352.2		26	1		0.19	0.57	90%	96%	98%
		414.2	254.1	x	30	1						
		414.2	94.1		56	3						
Heliotrine	15.98	314.2	156.1		30	1		0.24	0.71	104%	102%	83%
		314.2	138.1	x	20	4						
		314.2	94.1		39	2						
Heliotrine-N-oxide	18.90	330.2	172.1	x	28	4		0.25	0.76	102%	99%	89%
		330.2	111.1		47	2						
		330.2	80.1		66	2						
Integerrimine	19.93	336.2	138.1		32	1		0.62	1.9	81%	92%	108%
		336.2	120.1	x	30	4						
		336.2	94.1		36	2						
Integerrimine-N-oxide	21.71	352.2	136.1		36	1		0.65	2.0	97%	101%	81%
		352.2	118.1		36	5						
		352.2	94.1	x	42	4						
Intermedine	7.02	300.2	156.1		27	4		0.16	0.47	95%	96%	87%
		300.2	138.1		20	2						
		300.2	94.1	x	28	1						
Intermedine-N-oxide	10.79	316.2	172.1		30	4		0.34	1.0	104%	96%	74%
		316.2	138.1	x	32	4						
		316.2	94.1		52	1						
Jacobine	7.35	352.2	280.2		24	1		0.75	2.2	112%	104%	88%
		352.2	155.1	x	30	3						
		352.2	77.1		56	1						
Jacobine-N-oxide	10.59	368.2	296.2	x	24	1		0.45	1.4	99%	101%	84%
		368.2	120.1		40	4						
		368.2	94.1		58	3						
Lasiocarpine	26.49	412.2	336.2		19	2		0.24	0.73	93%	102%	87%
		412.2	220.1		19	3						
		412.2	120.1	x	27	1						
Lasiocarpine-N-oxide	27.30	428.2	352.2		24	1		0.64	1.9	98%	99%	104%
		428.2	254.2	x	32	1						
		428.2	94.1		54	1						
Lycopsamine	7.54	300.2	156.1		32	1		0.13	0.40	94%	97%	88%
		300.2	138.1		22	1						
		300.2	94.1	x	29	1						
Lycopsamine-N-oxide	11.49	316.2	172.1	x	30	4		0.25	0.74	101%	98%	72%
		316.2	138.1		30	4						
		316.2	94.1		48	1						
Monocrotaline	3.33	326.2	280.2		24	1		0.16	0.48	107%	101%	108%
		326.2	194.1		31	4						
		326.2	120.1	x	42	1						
Monocrotaline-N-oxide	7.06	342.2	137.1	x	33	2		0.72	2.2	102%	98%	63%
		342.2	120.1		39	1						
		342.2	94.1		56	2						
Retrorsine	14.17	352.2	138.1		31	2		0.87	2.6	95%	99%	86%
		352.2	120.1	x	32	1						
		352.2	94.1		36	1						
Retrorsine-N-oxide	16.88	368.2	136.1		42	1		2.2	6.5	97%	97%	81%
		368.2	118.1		33	1						
		368.2	94.1	x	52	2						
Riddelliine	9.69	350.2	138.1		32	2		2.6	7.9	101%	97%	89%
		350.2	120.1	x	32	3						
		350.2	94.1		44	2						
Riddelliine-N-oxide	12.33	366.2	136.1		38	2		3.6	11	101%	98%	84%
		366.2	120.1	x	38	3						
		366.2	94.1		50	2						
Rinderine-N-oxide	9.98	316.2	172.1	x	32	3	0.15		0.46	94%	95%	78%
		316.2	111.1		48	2						
		316.2	94.1		48	2						
Senecionine	20.31	336.2	138.1		34	1	0.17		0.51	98%	100%	106%
		336.2	120.1	x	30	2						
		336.2	94.1		40	1						
Senecionine-N-oxide	21.88	352.2	136.1		40	1	0.23		0.70	96%	98%	81%
		352.2	118.1		38	1						
		352.2	94.1	x	51	1						
Seneciphylline	15.67	334.2	138.1		29	2	0.82		2.5	106%	100%	88%
		334.2	120.1	x	30	3						
		334.2	94.1		40	2						
Seneciphylline-N-oxide	19.01	350.2	136.1		37	2	0.49		1.5	101%	102%	119%
		350.2	120.1		40	2						
		350.2	94.1	x	52	3						
Senecivernine	19.45	336.2	308.1		30	1	0.52		1.6	100%	104%	88%
		336.2	138.1		34	1						
		336.2	120.1	x	32	3						
Senecivernine-N-oxide	21.12	352.2	136.1		40	1	0.40		1.2	98%	99%	90%
		352.2	118.1		46	1						
		352.2	94.1	x	43	4						
Senkirkine	23.78	366.2	168.1	x	32	5	1.3		3.8	64%	90%	89%
		366.2	150.1		28	4						
		366.2	122.1		32	5						
Trichodesmine	12.90	354.2	308.2		24	2	0.76		2.3	95%	101%	88%
		354.2	222.1	x	32	2						
		354.2	121.1		35	1						

### Palynological analysis

The samples were brought up to room temperature, homogenized with a mortar. An aliquot of 100 mg homogenized bee pollen was weighed into a 50-mL tube (Buddeberg, Mannheim, Germany) and mixed with 10 mL demineralized water. After adding a drop of dish soap, the mixture was shaken by hand for 1 min. An aliquot of 10 µL was transferred to an object carrier, dried for 30 min, and covered with Kaiser’s glycerol gelatin for microscopy (Merck, Darmstadt, Germany). Three hundred pollen grains per sample were manually counted under a light microscope (10 × 40; VWR International, Darmstadt, Germany) which is considered representative for a pollen sample (Barth et al., 2012; Carpes et al., 2013; Morais et al., 2011). Due to the sample size of 57 pollen samples, 17,100 pollen grains were studied and subdivided into pollen from known PA producers and other plant families. Ten comparative samples were used to classify the pollen of PA-producing plants. The *Asteraceae* family includes many pollen types and each of them represents different plant species. However, pollen shape and size differ slightly from species to species within this family. For instance, the size of pollen was used to distinguish *Solidago* from *Senecio* pollen. Specifically, pollen sizes between 20 and 25 µm were assigned to pollen grains of the *Solidago*-type, which means all plants of *Asteraceae* which share similar shape and size of *Solidago* pollen cannot be differentiated. Therefore, *Eupatorium* sp*.* pollen were assigned to the group of *Solidago*-type. Pollen sizes > 30 µm (within the *Asteraceae* family) were considered to be of the *Senecio*-type. Using this classification scheme, *Petasites* and *Adenostyles* pollen belong to the *Senecio*-type. However, according to the literature, where measurements of six *Bidens* species and 20 *Senecio* species are listed, some *Bidens* species have similar pollen sizes as *Senecio* and vice versa (Beug, 2015). Hence, these two species could not be precisely distinguished by means of pollen sizes.

## Results and discussion

### Pyrrolizidine alkaloids detected by LC–MS/MS

**Fig. 2:**
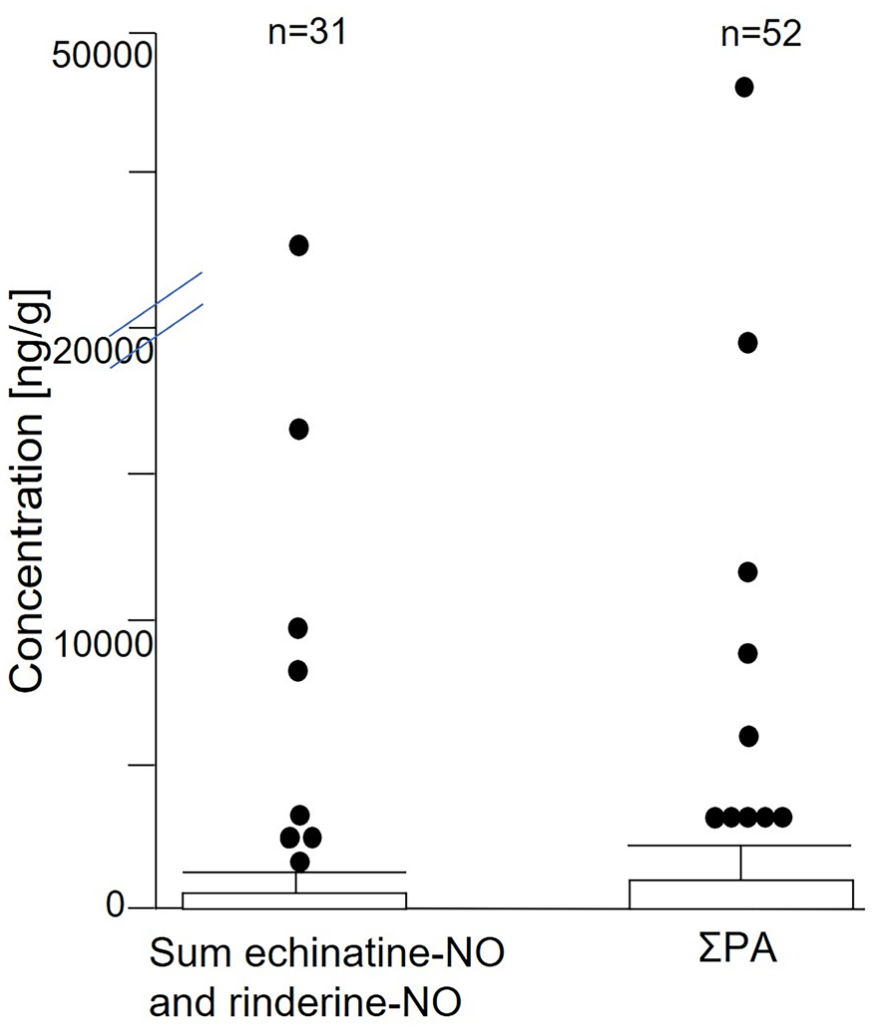
Boxplot chart (created with JMP® pro 15.0) showing the distribution of the sum of echinatine-NO and rinderine-NO next to the ΣPA concentrations (ng/g)

In total, 52 of the 57 analyzed samples were detected with positive PA findings. Except for three samples without detectable PA and two samples with levels below LOQ, ΣPA concentrations ranged from 0.48 to 48,400 ng/g bee pollen (Table 2; Fig. 2). Extraordinarily high ΣPA concentrations in a few samples had a strong impact on the mean values. This can be seen from the fact that median and mean ΣPA concentrations of 44 and 2160 ng/g bee pollen in all samples, respectively, varied by two orders of magnitude. The median and mean ΣPA concentrations in the positive samples showed only minimally higher values (Table 2).

**Table 2:** Main results of pyrrolizidine alkaloids (PA) analysis in pollen samples from 57 locations in Southern Germany sampled in July 2019

Positive samples	Minimum ∑PA conc (ng/g)*	Maximum ∑PA conc (ng/g)	Mean ∑PA conc in pos. samples (ng/g)	Median ∑PA conc in pos. samples (ng/g)	Mean ∑PA conc in all samples (ng/g)	Median ∑PA conc in all samples (ng/g)
52	0.48	48,400	2370	93.5	2160	44

^*^Seven samples (9%) were < LOD

Furthermore, 24 of the 42 analyzed PA/PANO were detected in the 57 samples (Table S3). This variety included 13 basic structures which were predominantly present as PANO (96%) along with minor contributions (4%) of all but two also as (free) PA (jacobine, retrorsine) and two of them as 7-acetyl-PA. As can be seen from Table 3, lycopsamine and intermedine are characteristic for plants of groups 2, 3, and 4, respectively, whereas plants of group 2 featured besides echimidine only lycopsamine and intermedine of the 42 PA included in the study.

**Table 3:** PA grouped to their botanical origin

Group	Pyrrolizidine alkaloid	Necine base	Esterification
Group 1 (*Senecio-*type)	Jacobine	Retronecine	Cyclic
	Retrorsine	Retronecine	Cyclic
	Seneciphylline	Retronecine	Cyclic
	Senecivernine	Retronecine	Cyclic
	Senecionine	Retronecine	Cyclic
	Erucifoline	Retronecine	Cyclic
	Integerrimine	Retronecine	Cyclic
Group 2 (*Echium-*type)	Echimidine	Retronecine	Di
	Lycopsamine	Retronecine	Mono
	Intermedine	Retronecine	Mono
Group 3 (*Borago-*type)	Lycopsamine	Retronecine	Mono
	Intermedine	Retronecine	Mono
Group 4 (*Eupatorium-*type)*	Lycopsamine	Retronecine	Mono
	Intermedine	Retronecine	Mono
	Echinatine with rinderine	Heliotridine	Mono

^*^Eupatorium sp. pollen is determined within the group of *Solidago*-/*Bidens*-type pollen

**Fig. 3:**
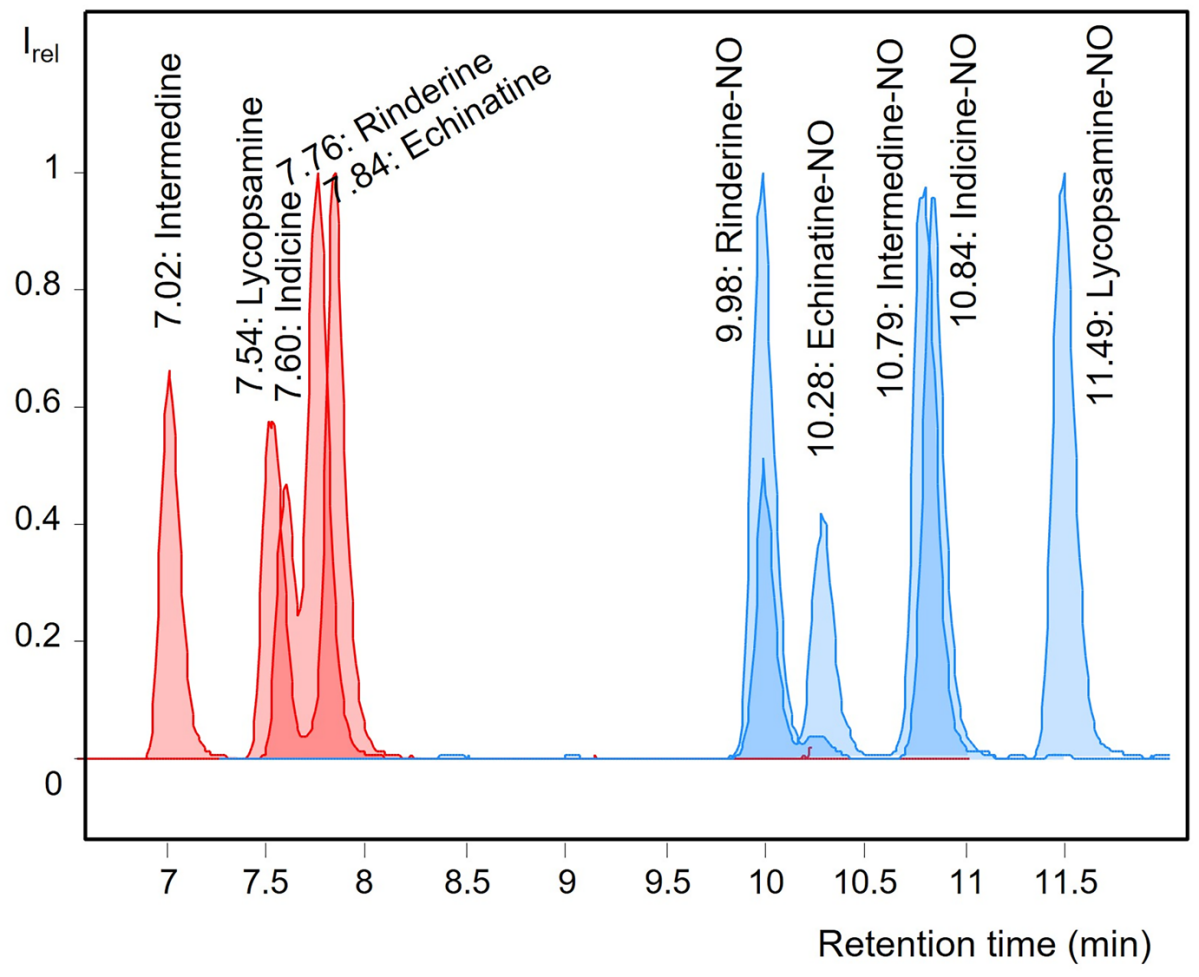
LC–MS/MS chromatogram (excerpt) showing the elution order of diastereomeric PA and PANO. Overlay of a pollen matrix sample spiked with PA calibration mix containing 42 PA(NO) and a standard solution containing rinderine, indicine, and their N-oxides

However, commercial bee pollen samples mostly consist of one pool sample collected over the whole collection season (April to August), while our samples were pooled within a short period of ~ 14 days (rainy days excluded) in the end of July. Compared to our short sampling period during the blooming time of PA producers, the longer collection periods over 5 months in the literature study may have caused a dilution effect by the inclusion of daily bee pollen samples free of PA. Although palynological analysis only indicated 33 samples with PA-containing pollen (Fig. S2), chemical LC–MS/MS analysis verified PA in 52 of 57 pollen samples (91%) (Fig. 3; Table S3). This could be partly due to a high number of screened PA as well as low LOD values of ~0.1–3.5 ng/g bee pollen. For instance, six out of 39 bee pollen samples containing echinatine featured this alkaloid at levels < 1 ng/g bee pollen (Table S3).

Altogether, 16 bee pollen samples exceeded ΣPA concentration of > 1000 ng/g bee pollen. Similarly, seven individual PANO and one PA (echinatine/rinderine, both co-eluted) individually contributed > 1000 ng/g bee pollen to ΣPA (Fig. S3). It is noteworthy that for the highest contaminated sample, echinatine-NO (23,900 ng/g bee pollen) and rinderine-NO (19,000 ng/g bee pollen) dominated the sample with free echinatine/rinderine at ~ 1000 ng/g bee pollen (Fig. 2). Hence, free echinatine/rinderine represented only ~ 2% of the PANO level. Also, in other samples, free echinatine/rinderine reached only 1–7% of the corresponding PANO level (sum of echinatine-NO and rinderine-NO) (Table 4; Table S3). This further illustrated the predominant role of PANO compared to PA. Apart from this group, lycopsamine-NO, intermedine-NO, echimidine-NO, retrorsine-NO, and also senecivernine-NO exceeded the 1000 ng/g bee pollen level in one or two samples (Fig. S3).

**Table 4:** PA pattern in pollen samples > 500 ng/g ΣPA compared to pollen counts

Nr	Concentration PA (ng/g)							Counted pollen			
	*Senecio*-type	Echimidine (-NO)	Echinatine (-NO)	Lycopsamine-NO	Intermedine-NO	Rinderine-NO	ΣPA [ng/g]	*Borago* sp.	*Echium* sp.	*Senecio* sp.	*Solidago/Bidens-Eupatorium*- type
47	110	0	24,900	2420	1920	19,000	48,400			97	37
30	65	0	9070	1220	1030	8040	19,400		20	10	
53	0	1	4550	690	955	5400	11,600				6
1	0	0	5000	305	170	3350	8830				6
43	9	0	3330	455	280	1900	5960			14	1
12	2880	3	390	30	10	80	3390				
45	5	0	1590	190	190	1220	3190			2	3
41	1540	0	715	120	120	665	3160			1	
44	1	0	1180	70	150	1740	3140		1		
15	410	0	1600	110	55	770	2950			2	164
25	2	2290	0	5	0	0	2300	3	53		
56	0	6	860	130	105	830	1930				9
16	0	0	760	205	105	360	1430				1
31	0	70	500	55	45	390	1060		2		
29	625	0	230	20	10	170	1050		1	1	2
14	25	0	570	115	60	260	1030			3	12
13	0	0	355	50	25	220	650				6
48	2	0	260	15	15	330	620				1
37	0	5	235	40	40	260	570				
55	420	0	100	4	1	30	550		1		1

**Fig. 4:**
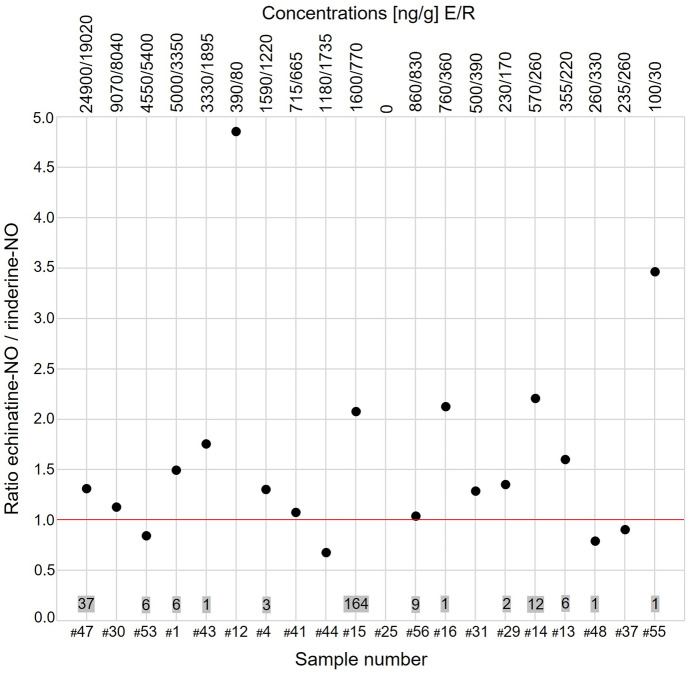
Point chart showing the ratio between echinatine (-NO) and rinderine-NO (E/R) with concentrations (ng/g) at the top and pollen counts of *Solidago-/Bidens-/Eupatorium*-type pollen at the bottom

Echinatine-NO (*n* = 8) and rinderine-NO (*n* = 7) not only show the highest frequency of > 1000 ng/g ΣPA concentrations, but both were usually detected with a very constant echinatine-NO/rinderine-NO (E/R) ratio of close to 1 (Fig. 4). This included all samples at ΣPA > 500 ng/g bee pollen that featured a high content of rinderine-NO compared to echinatine-NO. This produced strong evidence that both PA were co-occurring in the contaminated pollen and therefore originated from the same plant source. Interestingly, 70% of the samples featured both echinatine-NO and rinderine-NO on a similar level (except 6 samples; Table 3). These samples featured pollen of *Solidago-/Bidens-/Eupatorium*-type. As mentioned before, these genera could not be distinguished by palynological analysis (see “Materials and methods”). Despite the equivocal palynological verification, coincidence of *Eupatorium* sp.–type PA and potential *Eupatorium* sp.–type pollen was striking.

Hence, we propose that echinatine-NO and rinderine-NO, present in about the same ratio, are suitable markers for *Eupatorium-*type PA (Table 3, group 4). This classic distribution was modified by assigning echinatine-NO when predominant (absence of rinderine-NO) to group 2 (*Echium-*type) (Table 3). Likewise, both lycopsamine-NO and intermedine-NO are also belonging to groups 2, 3, and 4.

Co-occurrence of echinatine-NO and rinderine-NO (*Eupatorium*-type) was the common case (Fig. 4). Only sample #25 was highly contaminated with echimidine-NO (2290 ng ΣPA/g bee pollen), without remarkable amounts of rinderine-NO and other PANO characteristics for *Eupatorium*-type pollen (Fig. S4a, b). This pointed to the presence of *Echium-*type PA in sample #25, which was verified by palynological analysis. This was one of the few samples in which *Echium* sp. pollen was palynologically detected and also the one with the highest number of pollen of this kind (53 of 300 counts). However, high abundance of echimidine-NO in sample #25 also indicated contamination by *Borago* sp. pollen (3 counts) (Tables 3 and 4). One further sample (#31, two *Echium* sp. counts) featured echimidine-NO at 70 ng/g bee pollen. Extrapolation of the level in sample #31 from two to 53 pollen counts (70 ng/g multiplied by factor 26.5) would result in an echimidine-NO level of ~ 1860 ng/g bee pollen, which was very similar to the amount in sample #25. However, other samples with *Echium* sp. counts (#44, #31, #29, #55) were low in or did not feature echimidine(-NO). Apparently, this PA was unsuited as a reliable marker for *Echium* sp. and concentrations were comparably low (echivulgarine (-NO) was shown to be the main alkaloid for *Echium* sp. pollen (Dübecke et al., 2011)).

All our data supports that *Eupatorium* sp. (group 4) and not *Echium* sp. (group 2) was the predominant reason for high PA concentrations in the present samples, due to sample collection period end of July. However, as already discussed, the palynological detection of *Eupatorium* sp. was equivocal but mostly corresponding pollen was present when LC–MS/MS data indicated its presence. Based on this approach, 12 of the bee pollen samples with high PA content were assigned to group 4 (Fig. 4). Furthermore, most of these samples also featured lycopsamine-NO at around 10% of the concentration of rinderine-NO (Fig. S4a). For this reason, two samples also exceeded lycopsamine-NO levels of 1000 ng/g bee pollen ΣPA (Fig. 2).

As discussed above, most highly contaminated samples showed an E/R ratio close to 1 (Fig. 4). However, sample #12 formed an exception because it was richer in echinatine-NO (E/R ~ 4) and did not feature *Echium* sp. pollen. Instead, the *Senecio-*type PA (including senecivernine-NO and retrorsine-NO) were detected at much higher abundance and contributed the most with 2880 ng/g bee pollen to the considerably high load of PA (ΣPA 3390 ng/g bee pollen). However, pollen of *Senecio* sp. could not be detected either in this sample (Table 4). Also, both PA did not play any noticeable role in other pollen samples (Table S4). Furthermore, one sample (#41) showed an E/R ratio close to 1 but also high abundance of *Senecio*-type PA (including senecionine-NO and seneciphylline-NO) with 1530 ng/g bee pollen (Table S4). In this sample, *Senecio* sp. pollen was counted once. Also sample #29 showed 50% content of *Senecio*-type PA, but only one *Senecio* sp. pollen was counted (Table 4). In further six samples (#47, #30, #43, #45, #15, #14), *Senecio* sp. pollen were counted, but the *Senecio*-type PA content was comparably low (< 13%) as compared to ΣPA (Table 4).

Accordingly, no connection could be made between frequency of pollen counts and concentrations of individual PA or ΣPA in the samples. As already discussed, the percentage of pollen from known PA-producing plants was only 3% in the samples (Fig. S2) while PA concentrations could be extremely high. Therefore, in agreement with the literature (Kast et al., 2019), palynological analysis was not suited to identify samples with high PA load. However, the high ΣPA levels in several samples were alarming. For this reason, a risk assessment was performed.

### LC–MS/MS characteristics

The analyte spectrum (42 PA and PANO, see “Materials and methods”) comprised a number of isobaric substances not only showing the same nominal mass but also exhibiting high structural similarities and thus sometimes even identical or at least very similar MS/MS fragmentation patterns (Wuilloud et al., 2004). Therefore, chromatographic separation was granted special attention during method development to minimize possible co-elutions which bear the risk of erroneous peak identification. The use of a preferably long separation column in combination with a small particle size and a slow gradient elution profile (see “Materials and methods”) enabled chromatographic separation or at least partial separation of problematic compound groups such as the diastereomeric PA intermedine, lycopsamine, echinatine, rinderine, and indicine, as well as their N-oxides (Fig. 3). Because of potential interferences with lycopsamine and intermedine-NO, indicine(-NO) was not routinely included in the calibration standards. Likewise, echinatine was chosen over rinderine to be included in the calibration set as both PA were only partially resolved. In contrast, the method allowed both rinderine-NO and echinatine-NO to be quantitated simultaneously. Thus, samples containing rinderine had their rinderine levels determined as echinatine.

The presented LC–MS/MS method has to be understood as a target analysis technique. Therefore, individual PA(NO) that were not included in the calibration set may go undetected by this method. This especially applies to PA that were not yet commercially available as standards, unless they happen to be isobaric to analytes that were already part of the target method. To overcome this issue, non-target analysis, e.g., employing high-resolution mass spectrometry (LC-HRMS), could be performed if the corresponding more expensive instrumentation was on hand. In non-target mode, detection is not limited to a certain set of reference standards and therefore potentially covers all PA, regardless of their availability. However, an important advantage of target analysis using LC–MS/MS is its outstanding sensitivity, which is often superior to the sensitivity achieved with LC-HRMS systems. As the safe determination even of low PA(NO) levels was considered crucial for the intended purpose, minor drawbacks of the target analysis technique were thus deemed acceptable. Furthermore, regarding the separation problem of diastereomeric analytes, even HRMS would not have presented an improvement as interfering compounds are typically isobaric and thus pose the same problem regardless of the mass spectrometric resolving power.

### Palynological composition

Eighty-nine genera from 43 different plant families were identified by means of palynological inspection (Table S1), but only 3% (537 out of 17,100) of the inspected bee pollen grains could be traced back to PA-producing plants (Table 4; Fig. S2). Altogether, 33 samples (60%) contained pollen grains from known PA producers.

Pollen from *Echium* sp. were only present in seven of the present samples in this study (0.3% of all inspected pollen grains; Fig. S2). In addition, pollen of *Senecio* sp. were detected in 14 samples (1%) and *Borago* sp. in five samples (0.1%) (Fig. S2; Table S2). The highest share (1.7%) of PA-producing plants originated from *Solidago*-type and *Bidens*-type pollen (containing *Eupatorium* sp.) (23 samples) (Table S2).

### Result comparison to previous studies

The results of palynological analysis in this study showed a clear dominance of *Eupatorium* sp. pollen in the samples from the end of July (Fig. S2) while a similar study from Switzerland reported a clear dominance of *Echium* sp*.* among PA producers in pollen collected in June and a dominance of *Eupatorium* sp. from mid-July to August (Kast et al., 2018). The detected *Eupatorium* sp. pollen and the collection time by the end of July can be compared between the samples from Germany and Switzerland (Kast et al., 2018).

Predominance of PANO over free bases (96% vs. 4%; Table S3) agreed with other studies of PA-contaminated plant pollen (Bodi et al., 2014; Boppré et al., 2005; Dübecke et al., 2011). Also, the maximum ΣPA concentration of 48,400 ng/g bee pollen (#47) was only slightly higher than the top concentration reported before in commercial pollen samples (38,000 ng ΣPA/g bee pollen, Dübecke et al. (2011)). However, the detection frequency of PA (91% positive findings) in the present samples was higher compared to 30–60% positive findings in previous commercial bee pollen investigations. This could be partly due to a higher number of screened PA than in other studies (Dübecke et al., 2011; Kempf et al., 2010) as well as slightly lower LOD values obtained in the present study. Typical LOD of individual PA in other studies were 1–10 ng/g bee pollen (Dübecke et al., 2011; Kast et al., 2018; Kempf et al., 2010) compared to ~0.1–3.5 ng/g bee pollen in the present study.

Kast et al. (2018) analyzed one daily bee pollen sample on most of the weeks throughout the summers of 2012–2014 in Switzerland (one daily sample per week between April and September and flower heads from the neighborhood). This time series also indicated highest ΣPA concentrations in “*Eupatorium*-type PA” pollen samples collected at the end of July and the beginning of August (Kast et al., 2018). However, there was one remarkable difference between the results of Kast et al. (2018) and the present study. Although ΣPA concentrations were comparable between both studies, the PA pattern of *Eupatorium*-type pollen reported by Kast et al. (2018) was dominated by intermedine-NO and lycopsamine-NO. By contrast, both PANO were low concentrated in our samples which in turn were dominated by echinatine-NO and rinderine-NO (Table 4). Although all four PANO are known to occur in pollen of *Eupatorium* sp. plants (Roeder, 1995), the strikingly different PA patterns in the two studies from closely related regions were surprising. Since all four compounds were quantified in our samples by means of authentic reference standards (see “Materials and methods”), erroneous peak assignments could be excluded in our study. Compared to that, only a few PANO and none of the four crucial PANO were available to Kast et al. (2018) as reference standards. At this point, it is important to note that LC–MS/MS separation of intermedine-NO, rinderine-NO, echinatine-NO, and lycopsamine-NO was reported to be challenging (Colegate et al., 2012). Given the fact that rinderine-NO and echinatine-NO were not available as reference standards and a very short column (50 × 2.1 mm) was used by Kast et al. (2018), we concluded that the colleagues actually quantified these two PANO but wrongly labeled them as intermedine-NO and lycopsamine-NO although co-elutions of stereoisomers were discussed by the authors. Accordingly, a better resolution by LC–MS/MS and, most importantly, the availability of more reference standards in the present study enabled us to solve this discrepancy. Under this prerequisite, the highest concentrations of *Eupatorium*-type PA of 12,600 ng/g bee pollen reported by Kast et al. (2018) was on a similar level as top concentrations of 24,000 ng/g echinatine-NO of our samples.

### Risk assessment

**Fig. 5:**
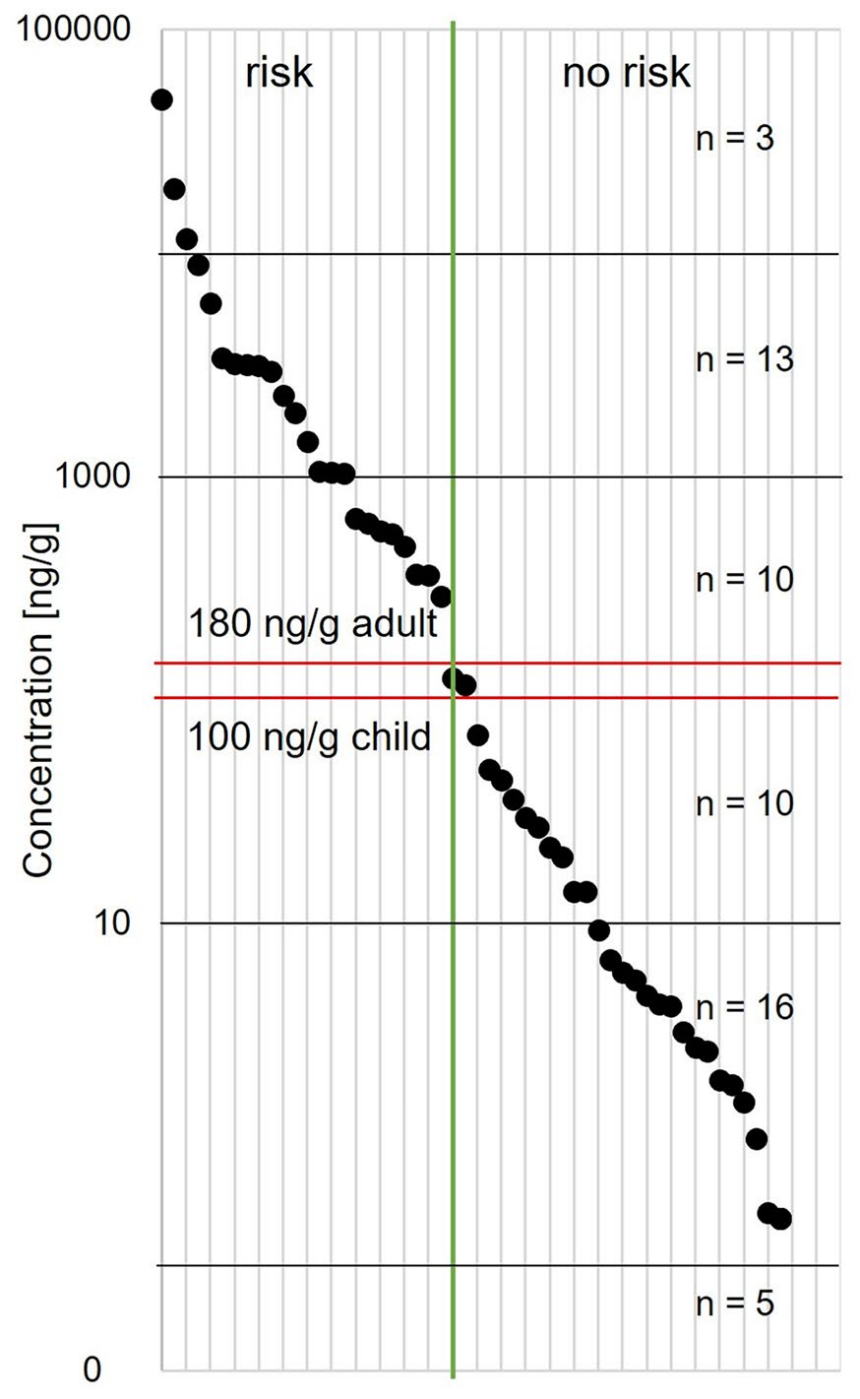
Point chart showing the ΣPA concentrations (ng/g) with a potential risk according to the BfR recommendations

To assess carcinogenic effects of PA, EFSA has established a reference point (EFSA, 2017), which results (by application of an MOE of 10,000) in a maximum recommended daily intake of 24 ng PA per kg BW and day for humans (BfR, 2020). Exposures below this dose are considered unlikely to cause deleterious effects. Typically, 5–10 g bee pollen (1–2 teaspoons) are consumed as food supplement per day. A general mean body weight of 75 kg and a daily intake of 10 g bee pollen result in a maximum recommended daily intake of 1800 ng ΣPA. Hence, all samples with ΣPA concentrations > 180 ng/g bee pollen exceeded the current dietary recommendations for adults. Accordingly, only 33 of the 57 bee pollen samples (58%) showed ΣPA concentrations below the target value. Namely, five samples featured no PA measurable concentration, 16 samples featured ΣPA levels between 0.5 and 10 ng/g, and 12 samples featured between 10 and 160 ng/g pollen. Two bee pollen samples of the latter group would already exceed the maximum recommended daily intake for children (10–11 years) due to the lower BW of 40 kg, resulting in a safe ΣPA level of only 100 ng/g bee pollen (Fig. 5) (GBE, 2017). By contrast, 24 samples (42%) exceeded the ΣPA target value of 180 ng/g pollen with eight samples between 170 and 1000 ng/g bee pollen. Remarkably, 13 samples were contaminated with 1000–10,000 ng ΣPA/g bee pollen and three samples even with > 10,000 ng ΣPA/g bee pollen which strongly exceeded the maximum recommended daily intake according to BfR based on a normal portion size (Fig. 5). This share was about twice as high as reported for commercial bee pollen samples from Switzerland, where one-third of commercial samples featured PA with seven samples exceeding the BfR recommendations with up to 1.185 ng/g ΣPA (Kast et al., 2018). However, the samples in our study were collected within 14 days in July, i.e., during the most prominent flowering period of PA-producing plants. In comparison, commercial samples are frequently pools of pollen harvested during several months, which may have a diluting effect on the PA pollen load. Hence, our study presents a worst-case scenario. However, this approach also made it possible to determine less abundant PA in the samples which might have been overlooked in other periods of the year. Moreover, 21% of the samples of the present study showed 10 to 270 times higher ΣPA concentrations than recommended (BfR, 2020). Also, preliminary relative potency factors (REPs) are used to assess the risk of PA exposure, the toxic effects, and the potencies of various congeners. For open-chain and cyclic diesters with *7S* configuration (e.g., lasiocarpine), REP values of 1.0 were calculated, and for monoesters with *7S* configuration (e.g., echinatine) values of 0.3, while open-chain diesters with *7R* configuration assigned values of 0.1 (e.g., echimidine) and *7R* monoesters 0.01 (e.g., intermedine) (Merz & Schrenk, 2016). The values of the N-oxides are related to the corresponding PA. Results of this study showed especially high values of echinatine-NO, rinderine-NO, and lycopsamine-NO where the REP values are comparably low. PA with high REP values could only be detected occasionally in high concentrations. In summary, the consumption of highly PA-contaminated bee pollen on a regular basis may pose an increased risk of cancer or liver disease and is therefore discouraged. In general, PA exposure should be kept as low as possible, especially since the total exposure toward PA may be influenced by additional PA sources in human nutrition such as herbal teas, spices, or honey.

## Conclusions

Ninety-one percent of the 57 bee pollen samples from different locations in Baden-Wuerttemberg featured PA with maximum ΣPA concentrations of 48,400 ng ΣPA/g bee pollen. The vast majority (96%) originated from PANO. Echinatine-NO and rinderine-NO showed highest concentrations of up to 23,900 ng/g bee pollen and were the most frequently detected PA in the samples collected at the end of July. About every third sample (20 out of 57 samples) exhibited ΣPA levels above the future legal maximum level of 500 ng/g pollen. In total, 24 pollen samples exceeded the BfR recommendation of 180 ng/g PA. Palynological analysis was found to be inappropriate to identify samples with high PA contamination.

The results of our study clearly demonstrate that almost half of the analyzed pollen samples from Southern Germany in July contained concentrations above the BfR recommendations. The occurrence of such high ΣPA concentrations could probably be minimized by stopping collecting bee pollen at the end of June (Kast et al., 2018). Furthermore, beekeepers in Southern Germany should avoid as much as possible the presence of *Eupatorium* sp. in the neighborhood around their hives. Bee pollen samples from Switzerland indicated no risk of contamination by pollen from *Eupatorium* sp. in months before July (Kast et al., 2018). Yet, further studies have to be conducted to check if this scenario can be assumed for Southern Germany as well. Also, commercial pollen samples from Germany should be examined for PA as they could consist pollen sampled in different months. At this point, bee pollen samples collected in July and before in Southern Germany should not be marketed or consumed without previous examination by means of LC–MS/MS analysis. The method applied in this study is suggested for application in order to establish a more robust assessment of PA content.

## Supplementary information

Below is the link to the electronic supplementary material.

Supplementary file1 (PDF 444 KB)

## Data Availability

The authors confirm the data generated or analyzed during this study are included in this published article and its supplementary files. All datasets are also available from the corresponding author on reasonable request.
