# Supplementary material for: High abundance of pyrrolizidine alkaloids in bee pollen collected in July 2019 from Southern Germany
Source: Environ Monit Assess. 2022 Mar 6;194(4):250. doi: 10.1007/s10661-022-09907-8 (PMC8898241; doi:10.1007/s10661-022-09907-8)

Supporting information in *Environmental Monitoring and Assessment* for:

High abundance of pyrrolizidine alkaloids in bee pollen collected in July 2019 from Southern Germany

Carolin Friedle[1*] · Thomas Kapp[2]· Klaus Wallner[1] · Raghdan Alkattea[1] · Walter Vetter[3]

[1]University of Hohenheim, Apicultural State Institute, Stuttgart, Germany

[2]Chemical and Veterinary Analysis Agency (CVUA) Stuttgart, Fellbach, Germany

[3]University of Hohenheim, Institute of Food Chemistry (170b), Stuttgart, Germany

*carolin_friedle@uni-hohenheim.de

Table S1 Detailed palynological analysis in all samples

Sample Number	*Aceraceae_Acer*	*Amaryllidaceae_Allium*	*Apiaceae*	*Apiaceae_Anthriscus-T*	*Apiaceae_Foeniculum*	*Apiaceae_Heracleum*	*Asparagaceae*	*Asparagaceae_Asparagus*	*Asteraceae_Achillea-T*	*Asteraceae_Arctium*	*Asteraceae_Artenmisia*	*Asteraceae_Bidens-T*	*Asteraceae_Calendula*	*Asteraceae_Carduus T*	*Asteraceae_Centaurea-T*	*Asteraceae_Centaurea*	*Asteraceae_Echinops*	*Asteraceae_Helianthus-T*
1									8			6		1				
2											3			3	3		6	34
3			1									1		2	37	70		5
4												1		8	24	3		199
5																57		14
6														4	41			53
7								128								30		42
8														1		7	3	
9																		
10																19		20
11						3										122		
12				3								1						
13									13					4	89	75		29
14													1					4
15										1				1	11	5		11
16						4			1					1		1		24
17				1					2							52		1
18																		
19		63													10	1		
20								8							1	2		
21									22					6		8		82
22									149							22		
23																2		
24							25							1				
25							10							6		26		9

Sample Number	Asteraceae_Senecio	Asteraceae_Solidago-T	Asteraceae_Taraxacum-T	Balsaminaceae_Impatiens	Boraginaceae_Borago	Boraginaceae_Echium	Boraginaceae_Phacelia	Brassicaceae	Brassicaceae_Alyssum	Brassicaceae_Brassica-T	Brassicaceae_Sinapis-T	Cannabaceae_Humulus	Caprifoliaceae_Knautia	Caprifoliaceae_Sambucus	Caprifoliaceae_Symphoricarpos	Caryophyllaceae	Chenopodiaceae	Clusiaceae_Hypericum
1			7				1											
2			6															
3							98											7
4				20			30			1								
5			4				169			1							1	
6			8				56											
7							49											1
8		17	5			1	59										1	
9			1				1							2				
10							86										1	65
11					1		52			2							1	
12			5								25	1					3	68
13		6			2		65											
14	3	12	14	2							15				1		5	10
15	2	164	2				15	3	30			1				1		5
16	1		10				51										2	20
17																		
18			6															13
19																		
20			1	3		1	8			114		3						12
21			30				13											6
22		2	6				71											
23							1											30
24			1								2						124	19
25			2		3	53	89			3								

Sample Number	*Convolvulaceae_Calystegia*	*Convolvulaceae_Convolvulus*	*Cornaceae_Cornus*	*Crassulaceae_Sedum*	*Cucurbitaceae_Cucurbita*	*Elaeagnaceae_Elaeagnus*	*Ericaceae_Calluna vulgaris*	*Euphorbiaceae_Mercurialis*	*Fabaceae_Gleditsia*	*Fabaceae_Lotus*	*Fabaceae_Melilotus-T*	*Fabaceae_Styphnolobium*	*Fabaceae_Trifolium pratense-T*	*Fabaceae_Trifolium repens-T*	*Fabaceae_Vicia-T*	*Fagaceae_Castanea sativa*	*Hydrangeaceae_Hydrangea*
1														4		118	1
2		4															
3													22	6			
4		3												3			
5														10			
6											5		28	34			
7														42			
8										16				6			
9																	
10									1				7	1			
11										1			6	35			
12		5												12			
13													4	6			
14														10			
15														8			
16									2		1			17			
17													239	3			
18											3			20	2	56	
19											2			32			
20										1			1	1		1	
21								2									
22																	
23						1								38			
24																	
25														15			

Sample Number	*Juglandaceae_Juglans*	*Lamiaceae_Lamium*	*Lamiaceae_Majoranus-T*	*Lamiaceae_Salvia-T*	*Lamiaceae_Teucrium*	*Lamiaceae_Thymus*	*Lythraceae_Lythrum*	*Malvaceae_Tilia*	*Onagraceae_Epilobium*	*Onagraceae_Oenothera*	*Orobanchaceae_Rhinanthus*	*Oxalidaceae_Oxalis*	*Papaveraceae*	*Papaveraceae_Corydalis*	*Papaveraceae_Eschscholzia californica*	*Papaveraceae_Papaver-T*	*Plantaginaceae_Plantago*	*Poaceae*
1											1	7					3	
2	4									1					5		37	
3																	8	
4			1														1	
5																		
6														62			9	
7															3		2	
8							2									3	75	7
9																1	137	
10																35	1	
11																	24	1
12										1						9	125	
13																	2	
14																	177	
15	3											2					24	
16																	118	1
17																	1	
18																1	1	2
19																	21	
20																	98	
21												2				28	65	
22								2								10	3	
23																	38	
24																	62	
25																28	2	

Sample Number	*Poaceae_Zea mays*	*Polemoniaceae_Phlox*	*Polygonaceae_Bistorta*	*Polygonaceae_Fagopyrum*	*Polygonaceae_Rumex*	*Ranunculaceae*	*Ranunculaceae_Clematis*	*Ranunculaceae_Nigella*	*Rosaceae*	*Rosaceae_Aruncus dioicus*	*Rosaceae_Filipendula*	*Rosaceae_Fragaria*	*Rosaceae_Potentilla*	*Rosaceae_Prunus-T*	*Rosaceae_Pyrus-T*	*Rosaceae_Rosa*	*Rosaceae_Rubus-T*	*Roseceae_Sanguisorba officinalis*
1	2					17					82						4	
2	1										1							
3																	5	
4																		
5	7			1														
6																		
7	1																	
8	7	2									22							
9	33						29				87					1		
10	29						23											
11	1										46						4	
12						1												
13	3			2														
14						19												
15													2					
16	2				1		1				39						2	
17		1																
18									6	11	12						46	
19	1										168							
20											33					6		
21	1					12												
22										2	19					5	7	
23	3										178						1	
24	15																	
25	9						4											

Sample Number	*Rutaceae_Tetradium*	*Scrophulariaceae_Buddlejeae*	*Scrophulariaceae_Verbascum*	*Simaroubaceae_Ailanthus altissima*	*Solanaceae*	*Tropaeolaceae_Tropaeolum*	*Urticaceae_Urtica*	*Vitaceae_Parthenocissus*	*Vitaceae_Vitis*
1					7			31	
2		4					1	187	
3								38	
4		6							
5		25						11	
6									
7								2	
8	2	43	1				4	16	
9		7						1	
10								12	
11							1		
12		33					8		
13									
14		9	1				15	2	
15		6					2	1	
16								1	
17									
18					121				
19							2		
20	2							4	
21		6						17	
22								2	
23			6					2	
24								51	
25								41	

Sample Number	*Aceraceae_Acer*	*Amaryllidaceae_Allium*	*Apiaceae*	*Apiaceae_Anthriscus-T*	*Apiaceae_Foeniculum*	*Apiaceae_Heracleum*	*Asparagaceae*	*Asparagaceae_Asparagus*	*Asteraceae_Achillea-T*	*Asteraceae_Arctium*	*Asteraceae_Artenmisia*	*Asteraceae_Bidens-T*	*Asteraceae_Calendula*	*Asteraceae_Carduus T*	*Asteraceae_Centaurea-T*	*Asteraceae_Centaurea*	*Asteraceae_Echinops*	*Asteraceae_Helianthus-T*
26														2				2
27	3								1					3				1
28									112									9
29									5					14	2			36
30														4	8			
31			2											1		68		1
32		3							9									
33															4			1
34				1		20									7	58		
35				2					2		62	2		3		43		63
36														5		4		1
37														1		41		33
38																		
39														6		71		7
40									3					1		59		1
41						2			2					1				
42			1												1	26		
43												1						
44				55		12			52					3		41	1	
45						2				1				1	5	7		
46														1				
47									1			37						
48					3										6	6		
49												1				47		4
50																		
51			1						1						5	1		
52			24															5
53		1	10						13					5		3		2
54														1		1		4
55												1						1
56									1			9			24	6	3	44
57												1		9		10		6

Sample Number	*Asteraceae_Senecio*	*Asteraceae_Solidago-T*	*Asteraceae_Taraxacum-T*	*Balsaminaceae_Impatiens*	*Boraginaceae_Borago*	*Boraginaceae_Echium*	*Boraginaceae_Phacelia*	*Brassicaceae*	*Brassicaceae_Alyssum*	*Brassicaceae_Brassica-T*	*Brassicaceae_Sinapis-T*	*Cannabaceae_Humulus*	*Caprifoliaceae_Knautia*	*Caprifoliaceae_Sambucus*	*Caprifoliaceae_Symphoricarpos*	*Caryophyllaceae*	*Chenopodiaceae*	*Clusiaceae_Hypericum*
26	1						236											
27		2	2							2						2		28
28										2							3	3
29	1	2				1												
30	20	10	3	1														1
31						2	102											
32			1	19													4	
33	1		20															
34		1	5	4			9						1					
35	15		4		11	6												
36			1				1										4	
37			12				38											6
38							7											
39			9		3		4								1			4
40			1				133											
41	1		10	4						6								67
42							67											
43	14		55	165			1											
44			4			1	2											7
45	2	3	7	8			9											9
46																		
47	97			48														
48																		
49			10	4			77											
50			4															
51							49											
52				14			10											2
53		6		10			22											1
54		3		16			7											12
55	1		39	14														
56				4														
57	4			65			28								2			

Sample Number	*Convolvulaceae_Calystegia*	*Convolvulaceae_Convolvulus*	*Cornaceae_Cornus*	*Crassulaceae_Sedum*	*Cucurbitaceae_Cucurbita*	*Elaeagnaceae_Elaeagnus*	*Ericaceae_Calluna vulgaris*	*Euphorbiaceae_Mercurialis*	*Fabaceae_Gleditsia*	*Fabaceae_Lotus*	*Fabaceae_Melilotus-T*	*Fabaceae_Styphnolobium*	*Fabaceae_Trifolium pratense-T*	*Fabaceae_Trifolium repens-T*	*Fabaceae_Vicia-T*	*Fagaceae_Castanea sativa*	*Hydrangeaceae_Hydrangea*
26													10	18			
27														1			3
28						1								9			
29											5			35			
30																	
31											3		10	11			
32													13	11			
33	1										1			15			
34											3		2	16			
35														20			
36			1										6	25			
37					2									86			
38		1												12			
39											11		4	62			
40													39	4			
41														1		92	
42											11		29	165			
43							3							1			
44										1	1			58			
45													40	74		1	
46										3				112			
47				6										7			
48													1	12			
49											14			137			
50													27	212			
51													210	9			
52											3		11	157	2		
53										15							
54														40			
55											3	6		61			
56																	
57														26			

Sample Number	*Juglandaceae_Juglans*	*Lamiaceae_Lamium*	*Lamiaceae_Majoranus-T*	*Lamiaceae_Salvia-T*	*Lamiaceae_Teucrium*	*Lamiaceae_Thymus*	*Lythraceae_Lythrum*	*Malvaceae_Tilia*	*Onagraceae_Epilobium*	*Onagraceae_Oenothera*	*Orobanchaceae_Rhinanthus*	*Oxalidaceae_Oxalis*	*Papaveraceae*	*Papaveraceae_Corydalis*	*Papaveraceae_Eschscholzia californica*	*Papaveraceae_Papaver-T*	*Plantaginaceae_Plantago*	*Poaceae*
26																10	10	
27											1				2		61	
28																	52	
29																	93	
30																	5	
31																	56	4
32																	114	
33																	153	17
34																	143	1
35						1									4	21	4	1
36																4	214	10
37																1	45	
38																	211	27
39																	34	
40																	31	
41		15			2											3	55	
42																		
43																	60	
44									1								36	
45																	64	
46		8														11	81	
47																	96	
48																	163	
49																	6	
50																	9	1
51																		
52		2															14	2
53				1			16										7	
54																2	1	
55																	109	
56																6	9	
57													64				21	

Sample Number	*Poaceae_Zea mays*	*Polemoniaceae_Phlox*	*Polygonaceae_Bistorta*	*Polygonaceae_Fagopyrum*	*Polygonaceae_Rumex*	*Ranunculaceae*	*Ranunculaceae_Clematis*	*Ranunculaceae_Nigella*	*Rosaceae*	*Rosaceae_Aruncus dioicus*	*Rosaceae_Filipendula*	*Rosaceae_Fragaria*	*Rosaceae_Potentilla*	*Rosaceae_Prunus-T*	*Rosaceae_Pyrus-T*	*Rosaceae_Rosa*	*Rosaceae_Rubus-T*	*Roseceae_Sanguisorba officinalis*
26							6				2							
27														17	29		9	
28	28										21							54
29	2										104							
30									38		142						2	
31	2										15							
32						3					120							
33	11										76							
34							3				24					2		
35	9		4		1			1								2		
36																		
37	3				1						15							
38	3					2					28						1	
39		1		44							38							
40	8										20							
41											35							
42																		
43																		
44		8														2	4	
45				5	2						37		23					
46											82							
47	1												2			1	2	
48	57						4					46						
49																		
50	3										44							
51	1						2				21							
52										4	30						17	
53	1	4								2	160							
54											46							
55						16											2	
56	3			67	2	54					26							
57	13				3						6							

Sample Number	*Rutaceae_Tetradium*	*Scrophulariaceae_Buddlejeae*	*Scrophulariaceae_Verbascum*	*Simaroubaceae_Ailanthus altissima*	*Solanaceae*	*Tropaeolaceae_Tropaeolum*	*Urticaceae_Urtica*	*Vitaceae_Parthenocissus*	*Vitaceae_Vitis*
26		2					1		
27	16	1					1	29	86
28	3			1				2	
29									
30					66				
31							5	18	
32							3		
33									
34									
35		1						16	
36								2	22
37								1	
38		1					6	1	
39			1						
40									
41								4	
42									
43									
44		10					1		
45									
46			2						
47								2	
48								2	
49									
50									
51									
52					3				
53		10					2	9	
54								167	
55		24				16	5	2	
56	4	7	2				1	28	
57								42	

Table S2 Palynological analysis of samples with pollen from PA-producing plants

Sample number	*Asteraceae_Senecio*	*Boraginaceae_Borago*	*Boraginaceae_Echium*	*Asteraceae_Bidens-T*	*Asteraceae_Solidago-T*
1				6	
3				1	
4				1	
8			1		17
11		1			
12				1	
13		2			6
14	3				12
15	2				164
16	1				
20			1		
22					2
25		3	53		
26	1				
27					2
29	1		1		2
30	20				10
31			2		
33	1				
34					1
35	15	11	6	2	
39		3			
41	1				
43	14			1	
44			1		
45	2				3
47	97			37	
49				1	
53					6
54					3
55	1			1	
56				9	
57	4			1	

Table S3 Detailed PA concentrations [ng/g] in all samples as obtained by the laboratory (CVUA)

Sample Number	Σ PA [ng/g]	7-Acetylintermedine	7-Acetylintermedine-N-oxide	7-Acetyllycopsamine	7-Acetyllycopsamine-N-oxide	Echimidine	Echimidine-N-oxide	Echinatine (with Rinderine)	Echinatine-N-oxide	Erucifoline	Erucifoline-N-oxide	Europine	Europine-N-oxide
1	8828	n.d.	n.d.	n.d.	n.d.	n.d.	n.d.	94	4910	n.d.	n.d.	n.d.	n.d.
2	14	n.d.	n.d.	n.d.	n.d.	n.d.	2.9	n.d.	n.d.	1.5	n.d.	n.d.	n.d.
3	2	n.d.	n.d.	n.d.	n.d.	n.d.	n.d.	1.5	n.d.	n.d.	n.d.	n.d.	n.d.
4	6.10	n.d.	n.d.	n.d.	n.d.	n.d.	n.d.	1.9	2.7	n.d.	n.d.	n.d.	n.d.
5	2.8	n.d.	n.d.	1.6	n.d.	n.d.	n.d.	n.d.	n.d.	n.d.	n.d.	n.d.	n.d.
6	4.4	n.d.	n.d.	n.d.	n.d.	n.d.	n.d.	1.6	1.6	n.d.	n.d.	n.d.	n.d.
7	2.7	n.d.	n.d.	n.d.	n.d.	1.6	1	n.d.	n.d.	n.d.	n.d.	n.d.	n.d.
8	363	n.d.	n.d.	n.d.	n.d.	5.8	7.5	4.2	261	n.d.	n.d.	n.d.	n.d.
9	1.9	n.d.	n.d.	n.d.	n.d.	1.4	n.d.	0.58	n.d.	n.d.	n.d.	n.d.	n.d.
10	117	n.d.	n.d.	n.d.	n.d.	16	78	1.1	3.1	2.8	n.d.	n.d.	n.d.
11	367	n.d.	n.d.	n.d.	n.d.	n.d.	n.d.	7.8	141	n.d.	n.d.	n.d.	n.d.
12	3388	n.d.	n.d.	n.d.	n.d.	1.8	1.6	9.7	379	n.d.	n.d.	n.d.	n.d.
13	650	n.d.	n.d.	n.d.	n.d.	n.d.	n.d.	8.7	345	n.d.	n.d.	n.d.	n.d.
14	1032	n.d.	n.d.	n.d.	n.d.	n.d.	n.d.	28	544	1.2	n.d.	n.d.	n.d.
15	2952	n.d.	n.d.	n.d.	n.d.	n.d.	n.d.	99	1502	26	329	n.d.	n.d.
16	1429	n.d.	n.d.	n.d.	n.d.	n.d.	n.d.	94	667	n.d.	n.d.	n.d.	n.d.
17	4.8	n.d.	n.d.	n.d.	n.d.	n.d.	n.d.	0.9	2.5	n.d.	n.d.	n.d.	n.d.
18	3.3	n.d.	n.d.	n.d.	n.d.	n.d.	3.3	n.d.	n.d.	n.d.	n.d.	n.d.	n.d.
19	20	n.d.	n.d.	n.d.	n.d.	n.d.	n.d.	2.1	9.8	n.d.	n.d.	n.d.	n.d.
20	22	n.d.	n.d.	n.d.	n.d.	n.d.	n.d.	2.1	7.8	2.6	n.d.	n.d.	n.d.
21	292	n.d.	n.d.	n.d.	n.d.	6.8	1.1	19	145	n.d.	n.d.	n.d.	n.d.
22	n.d.	n.d.	n.d.	n.d.	n.d.	n.d.	n.d.	n.d.	n.d.	n.d.	n.d.	n.d.	n.d.
23	4.3	n.d.	n.d.	n.d.	n.d.	n.d.	n.d.	0.74	n.d.	1.7	n.d.	n.d.	n.d.
24	27	n.d.	n.d.	n.d.	n.d.	n.d.	n.d.	1.5	3.3	n.d.	n.d.	n.d.	n.d.
25	2299	n.d.	n.d.	0.9	n.d.	173	2119	n.d.	n.d.	1.5	n.d.	n.d.	n.d.

Sample Number	Heliosupine	Heliosupine-N-oxide	Heliotrine	Heliotrine-N-oxide	Integerrimine	Integerrimine-N-oxide	Intermedine	Intermedine-N-oxide	Jacobine	Jacobine-N-oxide	Lasiocarpine	Lasiocarpine-N-oxide	Lycopsamine	Lycopsamine-N-oxide	Monocrotaline
1	n.d.	n.d.	n.d.	n.d.	n.d.	n.d.	4.7	166	n.d.	n.d.	n.d.	n.d.	1.4	302	n.d.
2	n.d.	n.d.	n.d.	n.d.	n.d.	n.d.	n.d.	4	n.d.	n.d.	n.d.	n.d.	n.d.	n.d.	n.d.
3	n.d.	n.d.	n.d.	n.d.	n.d.	n.d.	n.d.	n.d.	n.d.	n.d.	n.d.	n.d.	0.5	n.d.	n.d.
4	n.d.	n.d.	n.d.	n.d.	n.d.	n.d.	n.d.	n.d.	n.d.	n.d.	n.d.	n.d.	n.d.	n.d.	n.d.
5	n.d.	n.d.	n.d.	n.d.	n.d.	n.d.	n.d.	n.d.	n.d.	n.d.	n.d.	n.d.	1.1	n.d.	n.d.
6	n.d.	n.d.	n.d.	n.d.	n.d.	n.d.	n.d.	n.d.	n.d.	n.d.	n.d.	n.d.	n.d.	n.d.	n.d.
7	n.d.	n.d.	n.d.	n.d.	n.d.	n.d.	n.d.	n.d.	n.d.	n.d.	n.d.	n.d.	n.d.	n.d.	n.d.
8	n.d.	n.d.	n.d.	n.d.	n.d.	n.d.	n.d.	2.7	n.d.	n.d.	n.d.	n.d.	n.d.	2	n.d.
9	n.d.	n.d.	n.d.	n.d.	n.d.	n.d.	n.d.	n.d.	n.d.	n.d.	n.d.	n.d.	n.d.	n.d.	n.d.
10	n.d.	n.d.	n.d.	n.d.	n.d.	n.d.	n.d.	n.d.	n.d.	n.d.	n.d.	n.d.	0.4	n.d.	n.d.
11	n.d.	n.d.	n.d.	n.d.	n.d.	n.d.	1.4	23	n.d.	n.d.	n.d.	n.d.	1.2	20	n.d.
12	n.d.	n.d.	n.d.	n.d.	11	137	0.8	11	n.d.	n.d.	n.d.	n.d.	n.d.	28	n.d.
13	n.d.	n.d.	n.d.	n.d.	n.d.	n.d.	0.8	25	n.d.	n.d.	n.d.	n.d.	0.7	49	n.d.
14	n.d.	n.d.	n.d.	n.d.	n.d.	n.d.	2.2	59	n.d.	n.d.	n.d.	n.d.	2.8	111	n.d.
15	n.d.	n.d.	n.d.	n.d.	n.d.	12	2.8	52	n.d.	n.d.	n.d.	n.d.	4.3	108	n.d.
16	n.d.	n.d.	n.d.	n.d.	n.d.	n.d.	13	92	n.d.	n.d.	n.d.	n.d.	7.9	196	n.d.
17	n.d.	n.d.	n.d.	n.d.	n.d.	n.d.	n.d.	n.d.	n.d.	n.d.	n.d.	n.d.	n.d.	n.d.	n.d.
18	n.d.	n.d.	n.d.	n.d.	n.d.	n.d.	n.d.	n.d.	n.d.	n.d.	n.d.	n.d.	n.d.	n.d.	n.d.
19	n.d.	n.d.	n.d.	n.d.	n.d.	n.d.	n.d.	n.d.	n.d.	n.d.	n.d.	n.d.	n.d.	n.d.	n.d.
20	n.d.	n.d.	n.d.	n.d.	n.d.	n.d.	n.d.	n.d.	n.d.	n.d.	n.d.	n.d.	n.d.	n.d.	n.d.
21	n.d.	n.d.	n.d.	n.d.	n.d.	n.d.	0.9	6.9	n.d.	n.d.	n.d.	n.d.	0.9	9.3	n.d.
22	n.d.	n.d.	n.d.	n.d.	n.d.	n.d.	n.d.	n.d.	n.d.	n.d.	n.d.	n.d.	n.d.	n.d.	n.d.
23	n.d.	n.d.	n.d.	n.d.	n.d.	n.d.	n.d.	n.d.	n.d.	n.d.	n.d.	n.d.	n.d.	n.d.	n.d.
24	n.d.	n.d.	n.d.	n.d.	n.d.	n.d.	0.6	14	n.d.	n.d.	n.d.	n.d.	n.d.	1.6	n.d.
25	n.d.	n.d.	n.d.	n.d.	n.d.	n.d.	n.d.	n.d.	n.d.	n.d.	n.d.	n.d.	2.1	2.3	n.d.

Sample Number	Monocrotaline-N-oxide	Retrorsine	Retrorsine-N-oxide	Riddelliine	Riddelliine-N-oxide	Rinderine-N-oxide	Senecionine	Senecionine-N-oxide	Seneciphylline	Seneciphylline-N-oxide	Senecivernine	Senecivernine-N-oxide	Senkirkine	Trichodesmine
1	n.d.	n.d.	n.d.	n.d.	n.d.	3350	n.d.	n.d.	n.d.	n.d.	n.d.	n.d.	n.d.	n.d.
2	n.d.	n.d.	n.d.	n.d.	n.d.	n.d.	1.9	1.4	n.d.	2.3	n.d.	n.d.	n.d.	n.d.
3	n.d.	n.d.	n.d.	n.d.	n.d.	n.d.	n.d.	n.d.	n.d.	n.d.	n.d.	n.d.	n.d.	n.d.
4	n.d.	n.d.	n.d.	n.d.	n.d.	1.6	n.d.	n.d.	n.d.	n.d.	n.d.	n.d.	n.d.	n.d.
5	n.d.	n.d.	n.d.	n.d.	n.d.	n.d.	n.d.	n.d.	n.d.	n.d.	n.d.	n.d.	n.d.	n.d.
6	n.d.	n.d.	n.d.	n.d.	n.d.	1.1	n.d.	n.d.	n.d.	n.d.	n.d.	n.d.	n.d.	n.d.
7	n.d.	n.d.	n.d.	n.d.	n.d.	n.d.	n.d.	n.d.	n.d.	n.d.	n.d.	n.d.	n.d.	n.d.
8	n.d.	n.d.	n.d.	n.d.	n.d.	80	n.d.	n.d.	n.d.	n.d.	n.d.	n.d.	n.d.	n.d.
9	n.d.	n.d.	n.d.	n.d.	n.d.	n.d.	n.d.	n.d.	n.d.	n.d.	n.d.	n.d.	n.d.	n.d.
10	n.d.	2.7	n.d.	n.d.	n.d.	3.1	7	1.6	n.d.	1.6	n.d.	n.d.	n.d.	n.d.
11	n.d.	n.d.	n.d.	n.d.	n.d.	174	n.d.	n.d.	n.d.	n.d.	n.d.	n.d.	n.d.	n.d.
12	n.d.	68	1048	n.d.	n.d.	80	75	355	3.8	69	72	1037	n.d.	n.d.
13	n.d.	n.d.	n.d.	n.d.	n.d.	221	n.d.	n.d.	n.d.	n.d.	n.d.	n.d.	n.d.	n.d.
14	n.d.	7.3	n.d.	n.d.	n.d.	259	3.1	1	n.d.	n.d.	8.4	4.6	n.d.	n.d.
15	n.d.	n.d.	n.d.	n.d.	n.d.	771	15	12	n.d.	18	n.d.	n.d.	n.d.	n.d.
16	n.d.	n.d.	n.d.	n.d.	n.d.	358	n.d.	n.d.	n.d.	n.d.	n.d.	n.d.	n.d.	n.d.
17	n.d.	n.d.	n.d.	n.d.	n.d.	1.4	n.d.	n.d.	n.d.	n.d.	n.d.	n.d.	n.d.	n.d.
18	n.d.	n.d.	n.d.	n.d.	n.d.	n.d.	n.d.	n.d.	n.d.	n.d.	n.d.	n.d.	n.d.	n.d.
19	n.d.	n.d.	n.d.	n.d.	n.d.	8.3	n.d.	n.d.	n.d.	n.d.	n.d.	n.d.	n.d.	n.d.
20	n.d.	n.d.	n.d.	n.d.	n.d.	7.2	1	0.76	n.d.	n.d.	n.d.	n.d.	n.d.	n.d.
21	n.d.	n.d.	n.d.	n.d.	n.d.	102	n.d.	n.d.	n.d.	n.d.	n.d.	n.d.	n.d.	n.d.
22	n.d.	n.d.	n.d.	n.d.	n.d.	n.d.	n.d.	n.d.	n.d.	n.d.	n.d.	n.d.	n.d.	n.d.
23	n.d.	n.d.	n.d.	n.d.	n.d.	n.d.	n.d.	1.8	n.d.	n.d.	n.d.	n.d.	n.d.	n.d.
24	n.d.	n.d.	n.d.	n.d.	n.d.	3.1	1.1	2.1	n.d.	n.d.	n.d.	n.d.	n.d.	n.d.
25	n.d.	n.d.	n.d.	n.d.	n.d.	n.d.	n.d.	n.d.	n.d.	n.d.	n.d.	n.d.	n.d.	n.d.

Sample Number	Σ PA [µg/kg]	7-Acetylintermedine	7-Acetylintermedine-N-oxide	7-Acetyllycopsamine	7-Acetyllycopsamine-N-oxide	Echimidine	Echimidine-N-oxide	Echinatine (with Rinderine)	Echinatine-N-oxide	Erucifoline	Erucifoline-N-oxide	Europine	Europine-N-oxide
26	n.d.	n.d.	n.d.	n.d.	n.d.	n.d.	n.d.	n.d.	n.d.	n.d.	n.d.	n.d.	n.d.
27	1.6	n.d.	n.d.	n.d.	n.d.	n.d.	n.d.	n.d.	n.d.	n.d.	n.d.	n.d.	n.d.
28	70	n.d.	n.d.	n.d.	n.d.	n.d.	n.d.	n.d.	n.d.	2.3	55	n.d.	n.d.
29	1049	n.d.	n.d.	n.d.	n.d.	n.d.	n.d.	32	195	198	264	n.d.	n.d.
30	19426	n.d.	n.d.	n.d.	n.d.	n.d.	n.d.	607	8465	2.9	13	n.d.	n.d.
31	1056	n.d.	1.5	0.99	n.d.	16	53	22	476	n.d.	n.d.	n.d.	n.d.
32	n.d.	n.d.	n.d.	n.d.	n.d.	n.d.	n.d.	n.d.	n.d.	n.d.	n.d.	n.d.	n.d.
33	1.1	n.d.	n.d.	n.d.	n.d.	1.1	n.d.	n.d.	n.d.	n.d.	n.d.	n.d.	n.d.
34	6.9	n.d.	n.d.	n.d.	n.d.	n.d.	n.d.	n.d.	n.d.	1.1	5	n.d.	n.d.
35	44	n.d.	n.d.	1.3	n.d.	16	22	n.d.	n.d.	1.2	n.d.	n.d.	n.d.
36	9.4	n.d.	n.d.	n.d.	n.d.	n.d.	n.d.	1.7	n.d.	4.7	n.d.	n.d.	n.d.
37	573	n.d.	n.d.	n.d.	n.d.	1.6	3.6	6.6	228	n.d.	n.d.	n.d.	n.d.
38	n.d.	n.d.	n.d.	n.d.	n.d.	n.d.	n.d.	n.d.	n.d.	n.d.	n.d.	n.d.	n.d.
39	5.6	n.d.	n.d.	1.6	n.d.	n.d.	n.d.	0.67	n.d.	n.d.	n.d.	n.d.	n.d.
40	489	n.d.	n.d.	n.d.	n.d.	n.d.	n.d.	57	223	n.d.	n.d.	n.d.	n.d.
41	3159	n.d.	n.d.	n.d.	n.d.	n.d.	n.d.	50	665	11	350	n.d.	n.d.
42	0.48	n.d.	n.d.	n.d.	n.d.	n.d.	n.d.	n.d.	n.d.	n.d.	n.d.	n.d.	n.d.
43	5962	n.d.	n.d.	n.d.	n.d.	n.d.	n.d.	511	2814	0.93	n.d.	n.d.	n.d.
44	3133	n.d.	n.d.	n.d.	n.d.	n.d.	n.d.	86	1092	1	n.d.	n.d.	n.d.
45	3185	n.d.	n.d.	n.d.	n.d.	n.d.	n.d.	125	1460	n.d.	n.d.	n.d.	n.d.
46	14	n.d.	n.d.	n.d.	n.d.	n.d.	1.3	n.d.	n.d.	n.d.	n.d.	n.d.	n.d.
47	48395	n.d.	n.d.	n.d.	n.d.	n.d.	n.d.	1001	23933	2.9	18	n.d.	n.d.
48	621	n.d.	n.d.	n.d.	n.d.	n.d.	n.d.	16	244	n.d.	n.d.	n.d.	n.d.
49	0.51	n.d.	n.d.	n.d.	n.d.	n.d.	n.d.	0.51	n.d.	n.d.	n.d.	n.d.	n.d.
50	30	n.d.	n.d.	n.d.	n.d.	n.d.	n.d.	n.d.	n.d.	1.4	3.1	n.d.	n.d.
51	n.d.	n.d.	n.d.	n.d.	n.d.	n.d.	n.d.	n.d.	n.d.	n.d.	n.d.	n.d.	n.d.
52	49	n.d.	n.d.	n.d.	n.d.	n.d.	n.d.	1.7	n.d.	3.7	35	n.d.	n.d.
53	11592	n.d.	n.d.	n.d.	n.d.	n.d.	1.3	290	4260	n.d.	n.d.	n.d.	n.d.
54	125	n.d.	n.d.	n.d.	n.d.	7.6	10	3.5	43	n.d.	n.d.	n.d.	n.d.
55	552	n.d.	n.d.	n.d.	n.d.	n.d.	n.d.	12	85	49	284	n.d.	n.d.
56	1928	n.d.	n.d.	n.d.	n.d.	2	4.4	24	836	n.d.	n.d.	n.d.	n.d.
57	36	n.d.	n.d.	n.d.	n.d.	6	2.9	0.73	n.d.	n.d.	n.d.	n.d.	n.d.

Sample Number	Heliosupine	Heliosupine-N-oxide	Heliotrine	Heliotrine-N-oxide	Integerrimine	Integerrimine-N-oxide	Intermedine	Intermedine-N-oxide	Jacobine	Jacobine-N-oxide	Lasiocarpine	Lasiocarpine-N-oxide	Lycopsamine	Lycopsamine-N-oxide	Monocrotaline
26	n.d.	n.d.	n.d.	n.d.	n.d.	n.d.	n.d.	n.d.	n.d.	n.d.	n.d.	n.d.	n.d.	n.d.	n.d.
27	n.d.	n.d.	n.d.	n.d.	n.d.	n.d.	n.d.	1.6	n.d.	n.d.	n.d.	n.d.	n.d.	n.d.	n.d.
28	n.d.	n.d.	n.d.	n.d.	n.d.	n.d.	n.d.	n.d.	n.d.	n.d.	n.d.	n.d.	n.d.	n.d.	n.d.
29	n.d.	n.d.	n.d.	n.d.	9.9	9.8	1.1	11	n.d.	n.d.	n.d.	n.d.	1.1	16	n.d.
30	n.d.	n.d.	n.d.	n.d.	n.d.	5.6	70	961	n.d.	n.d.	n.d.	n.d.	25	1192	n.d.
31	n.d.	n.d.	n.d.	n.d.	n.d.	n.d.	2.7	42	n.d.	n.d.	n.d.	n.d.	2.5	52	n.d.
32	n.d.	n.d.	n.d.	n.d.	n.d.	n.d.	n.d.	n.d.	n.d.	n.d.	n.d.	n.d.	n.d.	n.d.	n.d.
33	n.d.	n.d.	n.d.	n.d.	n.d.	n.d.	n.d.	n.d.	n.d.	n.d.	n.d.	n.d.	n.d.	n.d.	n.d.
34	n.d.	n.d.	n.d.	n.d.	n.d.	n.d.	n.d.	n.d.	n.d.	n.d.	n.d.	n.d.	n.d.	n.d.	n.d.
35	n.d.	n.d.	n.d.	n.d.	n.d.	n.d.	0.8	n.d.	n.d.	n.d.	n.d.	n.d.	1.2	0.98	n.d.
36	n.d.	n.d.	n.d.	n.d.	n.d.	n.d.	n.d.	n.d.	n.d.	n.d.	n.d.	n.d.	n.d.	n.d.	n.d.
37	n.d.	n.d.	n.d.	n.d.	n.d.	n.d.	1	36	n.d.	n.d.	n.d.	n.d.	0.8	37	n.d.
38	n.d.	n.d.	n.d.	n.d.	n.d.	n.d.	n.d.	n.d.	n.d.	n.d.	n.d.	n.d.	n.d.	n.d.	n.d.
39	n.d.	n.d.	n.d.	n.d.	n.d.	n.d.	n.d.	n.d.	n.d.	n.d.	n.d.	n.d.	1.8	0.93	n.d.
40	n.d.	n.d.	n.d.	n.d.	n.d.	n.d.	3.6	17	n.d.	n.d.	n.d.	n.d.	2.8	22	n.d.
41	n.d.	n.d.	n.d.	n.d.	13	202	11	108	n.d.	5.8	n.d.	n.d.	3	117	n.d.
42	n.d.	n.d.	n.d.	n.d.	n.d.	n.d.	n.d.	n.d.	n.d.	n.d.	n.d.	n.d.	0.4	n.d.	n.d.
43	n.d.	n.d.	n.d.	n.d.	n.d.	n.d.	33	244	n.d.	n.d.	n.d.	n.d.	23	433	n.d.
44	n.d.	n.d.	n.d.	n.d.	n.d.	n.d.	4.8	143	n.d.	n.d.	n.d.	n.d.	1.7	69	n.d.
45	n.d.	n.d.	n.d.	n.d.	n.d.	n.d.	19	170	n.d.	n.d.	n.d.	n.d.	8.2	181	n.d.
46	n.d.	n.d.	n.d.	n.d.	n.d.	n.d.	n.d.	n.d.	n.d.	n.d.	n.d.	n.d.	n.d.	n.d.	n.d.
47	n.d.	n.d.	n.d.	n.d.	4.4	n.d.	92	1825	n.d.	n.d.	n.d.	n.d.	38	2378	n.d.
48	n.d.	n.d.	n.d.	n.d.	n.d.	n.d.	0.5	14	n.d.	n.d.	n.d.	n.d.	0.8	14	n.d.
49	n.d.	n.d.	n.d.	n.d.	n.d.	n.d.	n.d.	n.d.	n.d.	n.d.	n.d.	n.d.	n.d.	n.d.	n.d.
50	n.d.	n.d.	n.d.	n.d.	n.d.	2.8	n.d.	n.d.	n.d.	n.d.	n.d.	n.d.	n.d.	n.d.	n.d.
51	n.d.	n.d.	n.d.	n.d.	n.d.	n.d.	n.d.	n.d.	n.d.	n.d.	n.d.	n.d.	n.d.	n.d.	n.d.
52	n.d.	n.d.	n.d.	n.d.	n.d.	n.d.	n.d.	n.d.	n.d.	n.d.	n.d.	n.d.	n.d.	n.d.	n.d.
53	n.d.	n.d.	n.d.	n.d.	n.d.	n.d.	57	898	n.d.	n.d.	n.d.	n.d.	20	668	n.d.
54	n.d.	n.d.	n.d.	n.d.	n.d.	n.d.	0.8	13	n.d.	n.d.	n.d.	n.d.	0.8	12	n.d.
55	n.d.	n.d.	n.d.	n.d.	5.2	21	n.d.	1.3	n.d.	n.d.	n.d.	n.d.	0.5	3.8	n.d.
56	n.d.	n.d.	n.d.	n.d.	n.d.	n.d.	1.8	104	n.d.	n.d.	n.d.	n.d.	1.1	128	n.d.
57	n.d.	n.d.	n.d.	n.d.	n.d.	n.d.	0.6	22	n.d.	n.d.	n.d.	n.d.	n.d.	1.7	n.d.

Sample Number	Monocrotaline-N-oxide	Retrorsine	Retrorsine-N-oxide	Riddelliine	Riddelliine-N-oxide	Rinderine-N-oxide	Senecionine	Senecionine-N-oxide	Seneciphylline	Seneciphylline-N-oxide	Senecivernine	Senecivernine-N-oxide	Senkirkine	Trichodesmine
26	n.d.	n.d.	n.d.	n.d.	n.d.	n.d.	n.d.	n.d.	n.d.	n.d.	n.d.	n.d.	n.d.	n.d.
27	n.d.	n.d.	n.d.	n.d.	n.d.	n.d.	n.d.	n.d.	n.d.	n.d.	n.d.	n.d.	n.d.	n.d.
28	n.d.	n.d.	n.d.	n.d.	n.d.	n.d.	n.d.	6.4	n.d.	6.4	n.d.	n.d.	n.d.	n.d.
29	n.d.	n.d.	n.d.	n.d.	n.d.	168	51	15	35	42	n.d.	n.d.	n.d.	n.d.
30	n.d.	n.d.	n.d.	n.d.	n.d.	8041	4.9	13	4.2	21	n.d.	n.d.	n.d.	n.d.
31	n.d.	n.d.	n.d.	n.d.	n.d.	387	n.d.	n.d.	n.d.	n.d.	n.d.	n.d.	n.d.	n.d.
32	n.d.	n.d.	n.d.	n.d.	n.d.	n.d.	n.d.	n.d.	n.d.	n.d.	n.d.	n.d.	n.d.	n.d.
33	n.d.	n.d.	n.d.	n.d.	n.d.	n.d.	n.d.	n.d.	n.d.	n.d.	n.d.	n.d.	n.d.	n.d.
34	n.d.	n.d.	n.d.	n.d.	n.d.	n.d.	n.d.	0.84	n.d.	n.d.	n.d.	n.d.	n.d.	n.d.
35	n.d.	n.d.	n.d.	n.d.	n.d.	n.d.	n.d.	n.d.	n.d.	n.d.	n.d.	n.d.	n.d.	n.d.
36	n.d.	n.d.	n.d.	n.d.	n.d.	0.54	2.5	n.d.	n.d.	n.d.	n.d.	n.d.	n.d.	n.d.
37	n.d.	n.d.	n.d.	n.d.	n.d.	259	n.d.	n.d.	n.d.	n.d.	n.d.	n.d.	n.d.	n.d.
38	n.d.	n.d.	n.d.	n.d.	n.d.	n.d.	n.d.	n.d.	n.d.	n.d.	n.d.	n.d.	n.d.	n.d.
39	n.d.	n.d.	n.d.	n.d.	n.d.	0.6	n.d.	n.d.	n.d.	n.d.	n.d.	n.d.	n.d.	n.d.
40	n.d.	n.d.	n.d.	n.d.	n.d.	164	n.d.	n.d.	n.d.	n.d.	n.d.	n.d.	n.d.	n.d.
41	n.d.	n.d.	29	n.d.	n.d.	665	62	544	13	287	n.d.	16	n.d.	n.d.
42	n.d.	n.d.	n.d.	n.d.	n.d.	n.d.	n.d.	n.d.	n.d.	n.d.	n.d.	n.d.	n.d.	n.d.
43	n.d.	n.d.	n.d.	n.d.	n.d.	1895	5.9	2	n.d.	n.d.	n.d.	n.d.	n.d.	n.d.
44	n.d.	n.d.	n.d.	n.d.	n.d.	1735	n.d.	n.d.	n.d.	n.d.	n.d.	n.d.	n.d.	n.d.
45	n.d.	n.d.	n.d.	n.d.	n.d.	1217	0.59	3.1	n.d.	1.5	n.d.	n.d.	n.d.	n.d.
46	n.d.	n.d.	n.d.	n.d.	n.d.	n.d.	3.1	6.3	n.d.	3.1	n.d.	n.d.	n.d.	n.d.
47	n.d.	n.d.	n.d.	n.d.	n.d.	19020	22	41	3.6	16	n.d.	2.4	n.d.	n.d.
48	n.d.	n.d.	n.d.	n.d.	n.d.	329	2.1	n.d.	n.d.	n.d.	n.d.	n.d.	n.d.	n.d.
49	n.d.	n.d.	n.d.	n.d.	n.d.	n.d.	n.d.	n.d.	n.d.	n.d.	n.d.	n.d.	n.d.	n.d.
50	n.d.	n.d.	n.d.	n.d.	n.d.	n.d.	13	7.9	n.d.	2.1	n.d.	n.d.	n.d.	n.d.
51	n.d.	n.d.	n.d.	n.d.	n.d.	n.d.	n.d.	n.d.	n.d.	n.d.	n.d.	n.d.	n.d.	n.d.
52	n.d.	n.d.	n.d.	n.d.	n.d.	1	0.57	3.6	n.d.	3.3	n.d.	n.d.	n.d.	n.d.
53	n.d.	n.d.	n.d.	n.d.	n.d.	5398	n.d.	n.d.	n.d.	n.d.	n.d.	n.d.	n.d.	n.d.
54	n.d.	n.d.	n.d.	n.d.	n.d.	34	n.d.	n.d.	n.d.	n.d.	n.d.	n.d.	n.d.	n.d.
55	n.d.	n.d.	n.d.	n.d.	n.d.	28	13	18	5.9	26	n.d.	n.d.	n.d.	n.d.
56	n.d.	n.d.	n.d.	n.d.	n.d.	826	n.d.	n.d.	n.d.	n.d.	n.d.	n.d.	n.d.	n.d.
57	n.d.	n.d.	n.d.	n.d.	n.d.	n.d.	n.d.	2.5	n.d.	n.d.	n.d.	n.d.	n.d.	n.d.

Table S4 Detailed PA pattern in pollen samples > 500 ng/g ΣPA compared to pollen counts

Counted pollen	*Solidago/Bidens/Eupatorium* -type	37		6	6	1		3			164		9	1		2	12	6	1		1
	Senecio sp.	97	10			14		2	1		2					1	3				
	Echium sp.		20							1		53			2	1					1
	Borago sp.											3									
	ΣPA [ng/g]	48395	19426	11592	8828	5962	3388	3185	3159	3133	2952	2299	1928	1429	1056	1049	1032	650	621	573	552
Concentration PA [ng/g]	Rinderine-NO	19020	8041	5398	3350	1895	80	1217	665	1735	771		826	358	387	168	259	221	329	259	28
	Intermedine-NO	1825	961	898	166	244	11	170	108	143	52		104	92	42	11	59	25	14	36	1
	Intermedine	92	70	57	5	33	1	19	11	5	3		2	13	3	1	2	1	1	1	
	7-Acetylintermedine-NO														2						
	Lycopsamine-NO	2378	1192	668	302	433	28	181	117	69	108	2	128	196	52	16	111	49	14	37	4
	Lycopsamine	38	25	20	1	23		8	3	2	4	2	1	8	3	1	3	1	1	1	0
	7-Acetyllycopsamine											1			1						
	Echinatine-NO	23933	8465	4260	4910	2814	379	1460	665	1092	1502		836	667	476	195	544	345	244	228	85
	Echinatine (incl. Rinderine)	1001	607	290	94	511	10	125	50	86	99		24	94	22	32	28	9	16	7	12
	Echimidine-NO			1			2					2119	4		53					4	
	Echimidine						2					173	2		16					2	
	Integerrimine-NO		6				137		202		12					10					21
	Integerrimine	4					11		13							10					5
	Erucifoline-NO	18	13						350		329					264					284
	Erucifoline	3	3			1			11	1	26	2				198	1				49
	Senecionine-NO	41	13			2	355	3	544		12					15	1				18
	Senecionine	22	5			6	75	1	62		15					51	3		2		13
	Senecivernine-NO	2					1037		16								5				
	Senecivernine						72										8				
	Seneciphylline-NO	16	21				69	2	287		18					42					26
	Seneciphylline	4	4				4		13							35					6
	Retrorsine-NO						1048		29												
	Retrorsine						68										7				
	Jacobine-NO								6												
	Nr	47	30	53	1	43	12	45	41	44	15	25	56	16	31	29	14	13	48	37	55

Figure S1 Location area of sampling in Baden-Wuerttemberg, Southern Germany (Mapping with JMP® pro 15.0; basic data © State Office for Geoinformation and Rural Development Baden-Wuerttemberg (www.lgl-bw.de) and from the Environmental Information System (UIS) of the LUBW State Institute for the Environment Baden-Wuerttemberg)

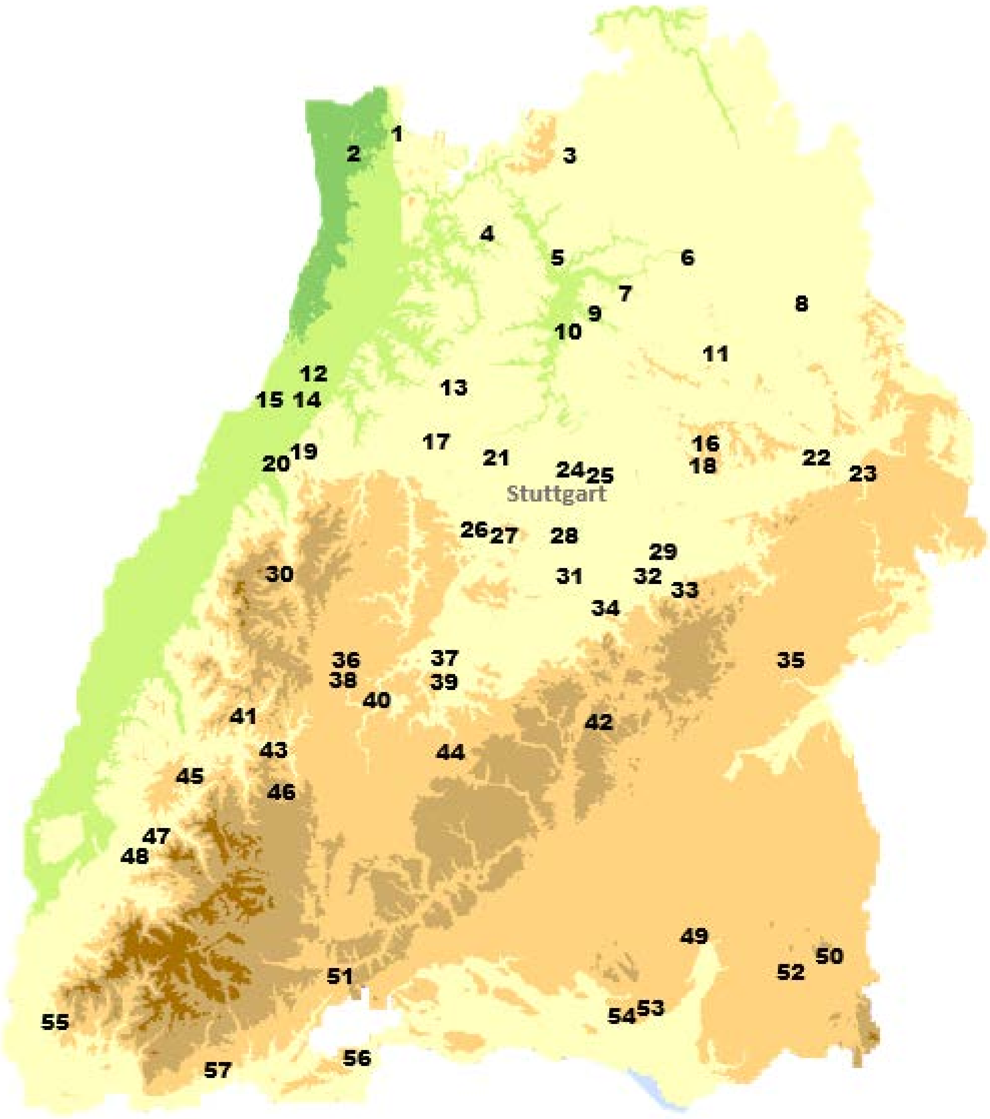

Figure S2 Composition of pollen counts from PA-producing plants

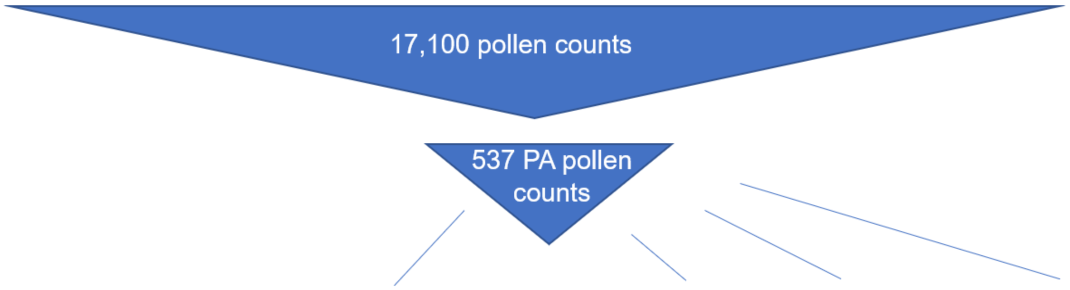

	Senecio sp.	*Echium* sp.	*Borago* sp.	*Solidago-/Bidens-/Eupatorium*-type
In total	163	65	20	289
% of total	1	0.1	0.1	1.7
% of PA plants	30	12	4	54
In samples	14	7	5	23
Max. counts per sample	97	53	11	164

*individual samples may contain more than one type of pollen

Figure S3 Bar chart showing the detection frequency of PA(NO) with concentrations > 1,000 ng/g

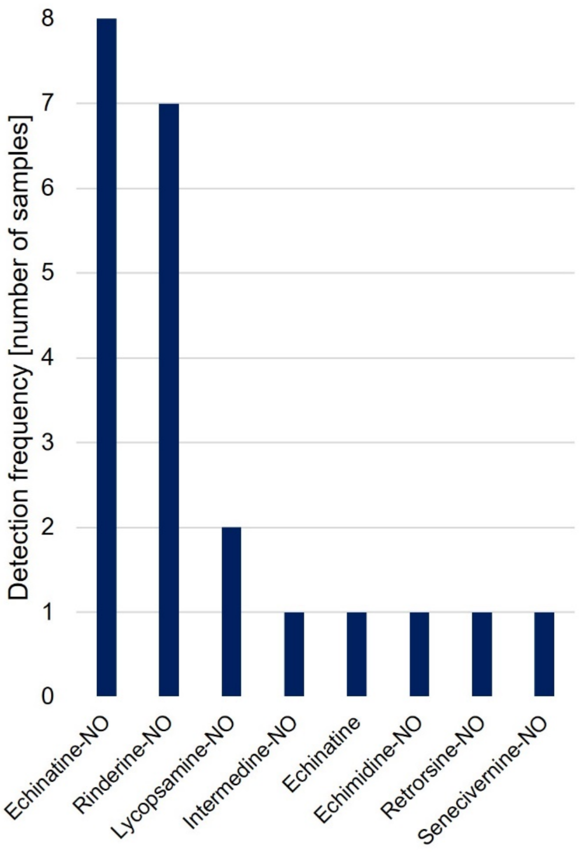

Figure S4 Point chart showing the ratio between (**a**) (7-acetyl-) lycopsamine-NO and rinderine-NO (L/R) with concentrations [ng/g] at the top (**b**) echimidine(-NO) and echinatine(-NO) (EM/E) with concentrations [ng/g] at the top and pollen counts of *Echium* sp. pollen at the bottom

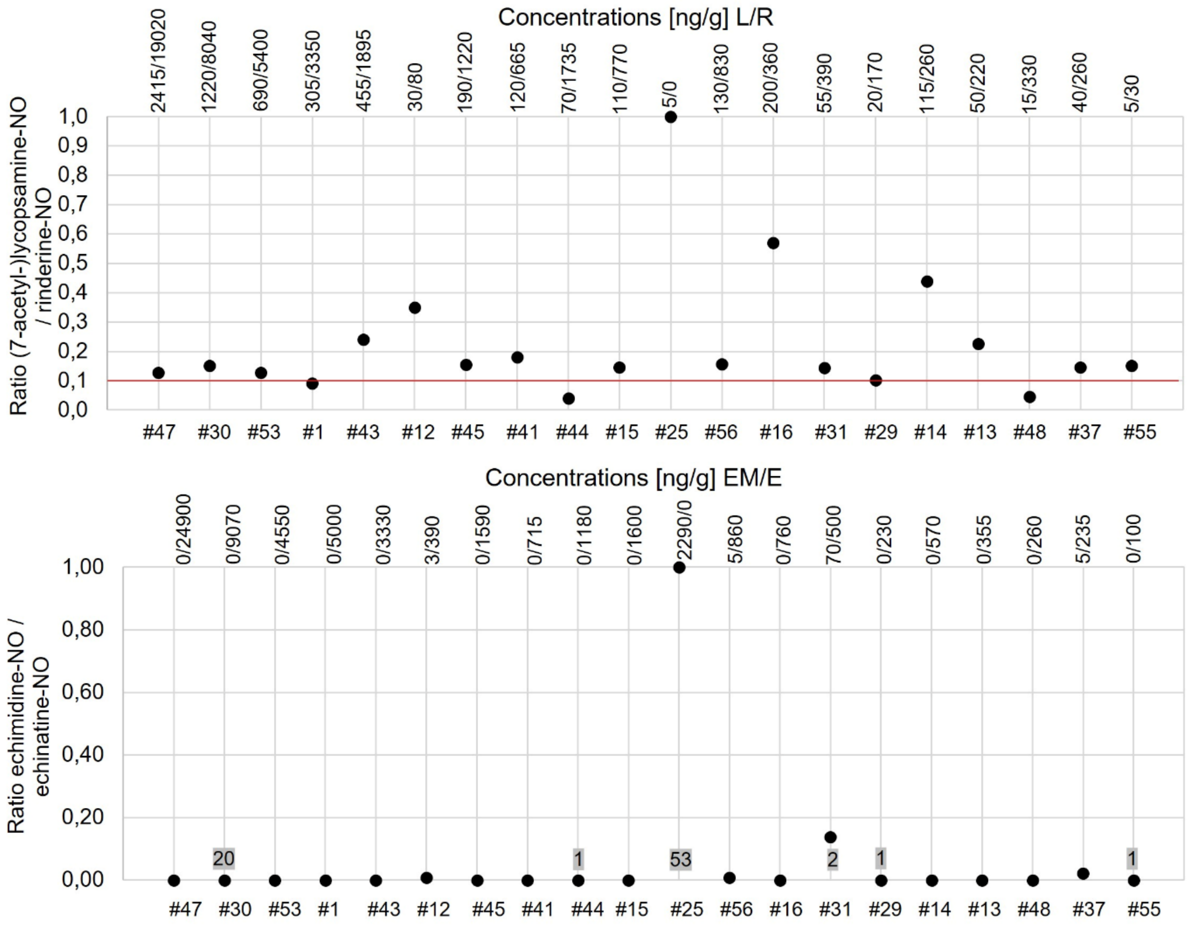

Supplement: Supplementary file 1 — Supplementary file1 (PDF 444 KB) [file 10661_2022_9907_MOESM1_ESM.pdf]
